# Exercise and mitochondrial mechanisms in patients with sarcopenia

**DOI:** 10.3389/fphys.2022.1040381

**Published:** 2022-12-06

**Authors:** Hamed Alizadeh Pahlavani, Ismail Laher, Beat Knechtle, Hassane Zouhal

**Affiliations:** ^1^ Department of Physical Education, Farhangian University, Tehran, Iran; ^2^ Department of Anesthesiology, Pharmacology, and Therapeutics, Faculty of Medicine, University of British Columbia, Vancouver, BC, Canada; ^3^ Institute of Primary Care, University of Zurich, Zurich, Switzerland; ^4^ Medbase St Gallen Am Vadianplatz, St. Gallen, Switzerland; ^5^ Movement Sport, Health and Sciences Laboratory (M2S) UFR-STAPS, University of Rennes 2-ENS Cachan, Charles Tillon, France; ^6^ Institut International des Sciences Du Sport (2IS), Irodouer, France

**Keywords:** exercise, sarcopenia, mitochondria, mechanism, aging

## Abstract

Sarcopenia is a severe loss of muscle mass and functional decline during aging that can lead to reduced quality of life, limited patient independence, and increased risk of falls. The causes of sarcopenia include inactivity, oxidant production, reduction of antioxidant defense, disruption of mitochondrial activity, disruption of mitophagy, and change in mitochondrial biogenesis. There is evidence that mitochondrial dysfunction is an important cause of sarcopenia. Oxidative stress and reduction of antioxidant defenses in mitochondria form a vicious cycle that leads to the intensification of mitochondrial separation, suppression of mitochondrial fusion/fission, inhibition of electron transport chain, reduction of ATP production, an increase of mitochondrial DNA damage, and mitochondrial biogenesis disorder. On the other hand, exercise adds to the healthy mitochondrial network by increasing markers of mitochondrial fusion and fission, and transforms defective mitochondria into efficient mitochondria. Sarcopenia also leads to a decrease in mitochondrial dynamics, mitophagy markers, and mitochondrial network efficiency by increasing the level of ROS and apoptosis. In contrast, exercise increases mitochondrial biogenesis by activating genes affected by PGC1-ɑ (such as CaMK, AMPK, MAPKs) and altering cellular calcium, ATP-AMP ratio, and cellular stress. Activation of PGC1-ɑ also regulates transcription factors (such as TFAM, MEFs, and NRFs) and leads to the formation of new mitochondrial networks. Hence, moderate-intensity exercise can be used as a non-invasive treatment for sarcopenia by activating pathways that regulate the mitochondrial network in skeletal muscle.

## Introduction

Sarcopenia refers to a loss of muscle mass associated with aging that decreases mobility, independence, and the quality of life in the elderly (Liu et al., 2021). Sarcopenia ranks second to osteoporosis for degenerative diseases afflicting the elderly according to the World Health Organization (WHO) international classification of diseases in 2016 (Organization, 2020). Decreased muscle mass starts in the fifth decade of life (∼1% reduction per year) and accelerates in the seventh decade of life, eventually reducing muscle mass by about 30%–50%. Muscle weakness in sarcopenia increases the risk of age-related diseases such as cardiovascular disease, obesity, cancer, diabetes, and mortality (Romanello, 2021).

Sarcopenia occurs in 5%–13% of people over 60 years and in 50% of people over 80 years (Romanello, 2021). Skeletal muscle mass loss is greater in 60-year-old men than in women of the same age, while sarcopenia is present in approximately 53% of men compared with 47% of women. The prevalence of sarcopenia is 31% in women and 53% in men in people aged 80 years or more (Cesare et al., 2020). Sarcopenia is a disease related to aging and inactivity and is a strong predictor of disability, disease, and mortality in the elderly (Phu et al., 2015). The causes of sarcopenia include a sedentary lifestyle (Liu et al., 2021), reduced energy intake, reduced protein consumption, myocellular changes (ATP and glycogen reduction) (Dozio et al., 2021), mitochondrial dysfunction, mitochondrial biogenesis disorder, inappropriate mitochondrial dynamics, defects in mitochondrial turnover, mitochondrial depletion, excessive oxidative stress, increased inflammatory cytokines such as tumor necrosis factor-α (TNF-α) and interleukin-6 and -1α (Liang et al., 2021; Romanello, 2021). There is also much evidence to support a key role for mitochondrial dysfunction (Ferri et al., 2020).

Treatment options for sarcopenia include gene therapy, nutritional supplements, physical activity, anabolic hormones, endurance and resistance training, anti-inflammatory medications, and antioxidants (Jensen, 2008). Among these options, exercise is a low-cost and non-invasive (gene therapy) method. Exercise stimulates mitochondrial function, improves muscle metabolism, and regulates redox imbalance, muscle mitochondrial dysfunction, mitophagy, mitochondrial biogenesis, and apoptosis of muscle cells (Picca and Calvani, 2021). Hence, we review the effects of exercise on sarcopenia and discuss the changes in mitochondrial adaptations due to changes in oxidative stress, mitochondrial DNA (mtDNA) damage, antioxidant defense mechanisms, mitochondrial dynamics, mitophagy process, and mitochondrial biogenesis in skeletal muscle.

## Mitochondrial plasticity and oxidative stress in sarcopenia

**FIGURE 1:**
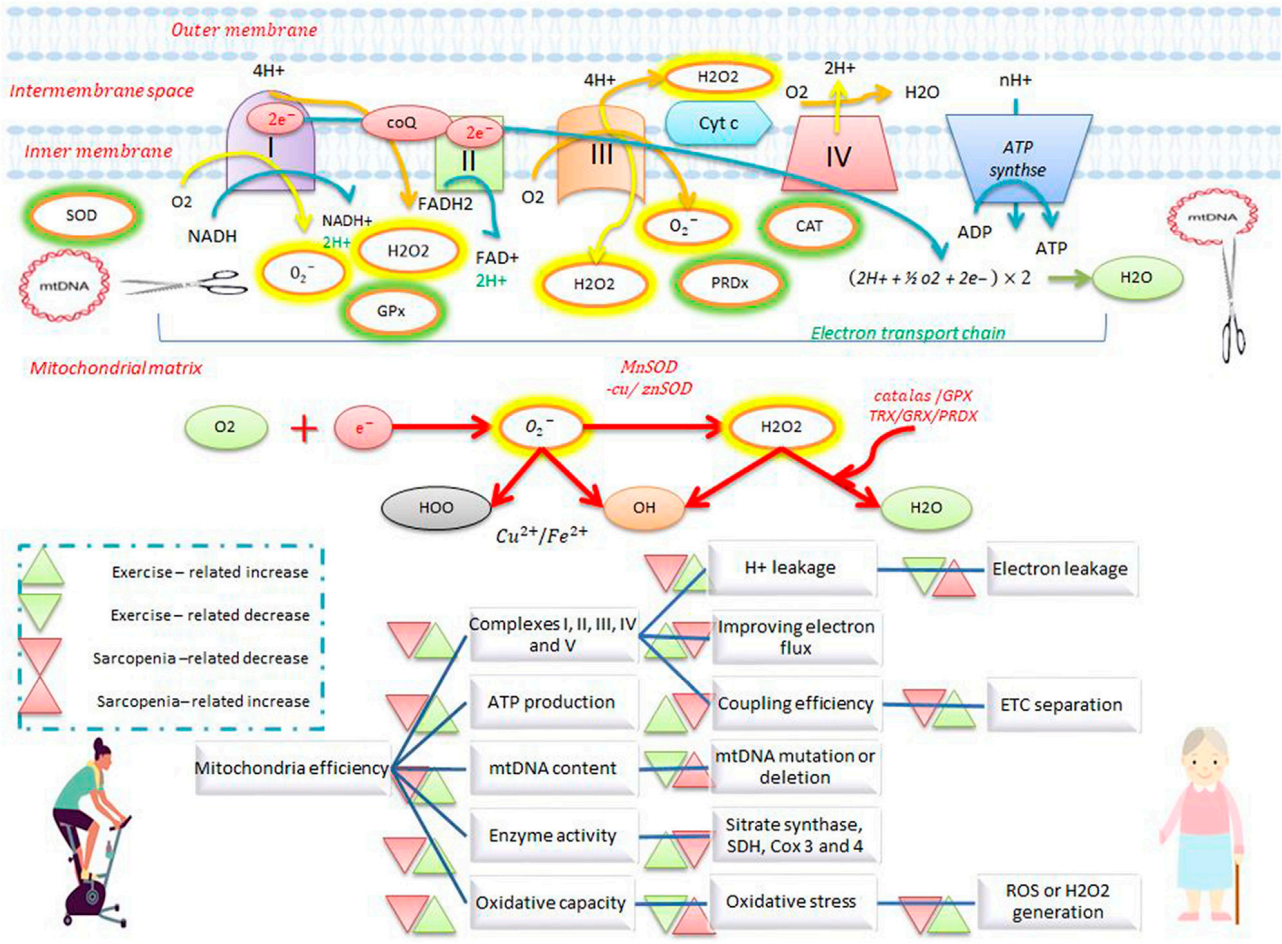
Electron transfer chain system and antioxidant defense during exercise and sarcopenia. Exercise increases the activity of complexes I, II, III, IV, and V, ATP production, enzymatic activity, oxidative capacity, and mtDNA repair, and reduces electron leakage, electron transfer chain separation, and ROS production. Exercise also stimulates antioxidant defenses by increasing antioxidants such as catalase, superoxide dismutase, and peroxiredoxin. Exercise opposes the effects of sarcopenia on mitochondrial activity and antioxidant defense.

Oxidative phosphorylation (OXPHOS) in mitochondria generates the ATP needed for various cellular processes whereby nicotinamide adenine dinucleotide (NADH) and flavin adenine dinucleotide (FADH2) transfer electrons to the electron transport chain (ETC). The ETC is a four-complex enzyme system for sequential oxidation and transfer of electrons, so that as electrons move from complex I to IV, protons are actively transported to the mitochondrial intermembrane space by complexes I, III, and IV. This movement of ions forms an electrochemical gradient between the intermembrane space and the matrix, where the matrix is negatively charged while the intermembrane space is positively charged. Adenosine diphosphate (ADP) is phosphorylated to adenosine triphosphate (ATP) when protons flow into the matrix *via* ATP synthase (V complex). The amount of ATP synthesized depends on the ETC flux as well as the amount of oxygen absorbed (Harper et al., 2021). In resting skeletal muscle, the concentration of intracellular ADP is lower than that of ATP, which suppresses OXPHOS activity in mitochondria and increases the NADH/NAD + ratio. This reduction in pressure can increase the leakage of unpaired electrons from the ETC (due to the flow of surplus electrons) into the ubiquinone (Q) complex. The reduction of cellular oxygen by electrons forms superoxide anions ( $O_{2}^{-}$ ), which are then spontaneously by SOD converted to H_2_O_2_ (Mason et al., 2020) (Figure 1). The main sites for the *in vitro* production of $O_{2}^{-}$ include the I-ubiquinone reducing site (∼23% of total $O_{2}^{-}$ ), the flavin site (∼20%), the flavin in complex II (24%), the complex III-ubiquinol oxidizing site (∼15%), and the fatty acid beta-oxidation pathway (EF ∼ 13%) (Figure 1) (Mason et al., 2020). With the onset of muscle contraction, ATP hydrolysis increases 100-fold relative to rest, and mitochondrial OXPHOS is readjusted due to changes in the ADP/ATP ratio. A rapid decrease in the flux of the mitochondrial ETC leads to a decrease in $O_{2}^{-}$ production. Furthermore, when ADP is used to stimulate OXPHOS, mitochondrial $O_{2}^{-}$ /H_2_O_2_ emission is dramatically reduced (Mason et al., 2020).

Resting and active muscles produce ROS such as $O_{2}^{-}$ , H_2_O_2_, and hydroxyl radicals (•OH) (Cesare et al., 2020). Furthermore, other ROS produced in mitochondria are generated primarily at complexes I, II, and III during normal respiration. Complex IV, as the final acceptor of ETC electrons, plays a significant role in reducing electron leakage and ROS production by increasing its activity (Lo et al., 2020). Increased ROS production during aging disrupts the components of the mitochondrial respiratory chain (MRC), which in turn further increases the production of free radicals, mitochondrial damage, and inactive mitochondria (Guescini et al., 2017). The defective performance of MRC enzyme complexes reduces the bioenergetic storage capacity and increases electron leakage from complexes I, II, and III (Guescini et al., 2017). Skeletal muscle mitochondria produce 50%–80% more H_2_O_2_ in older mice (28–29 months) than in younger mice (6–8 months), and moreover, muscle fibers from older mice have fewer copies of mtDNA than younger mice (Harper et al., 2021; Harper et al., 2021). In addition, increased ROS levels cause a further reduction in function and muscle mass due to inactivity (Waltz et al., 2018), and high levels of oxidative stress exacerbate ROS-induced sarcopenia (Sullivan-Gunn and Lewandowski, 2013; Corsetto et al., 2019). The respiratory chain is the main site for ROS production, and the proximity of the mitochondrial genome to the membrane facilitates ROS-induced mutations of mtDNA (Lo et al., 2020) (Figure 1). Aging causes oxidative damage to proteins, fats, and mtDNA. These injuries disrupt the normal functioning of ATP-producing mitochondria and lead to low energy production, increased mtDNA deletion, decreased enzyme activity, and increased oxidative stress. In sarcopenic aging, mtDNA mutations reduce the activity of citrate synthase (CS), COX, and succinate dehydrogenase (SDH) enzymes, which in turn reduces the activities of complexes I, II, III, V, and IV (44%–51%). There is a 30% reduction in ATP production in older mice (Marzetti et al., 2008; Migliavacca et al., 2019; Harper et al., 2021), and ETS abnormalities in sarcopenic muscle are also affected by decreases in muscle fibers (Marzetti et al., 2008). The production of mitochondrial free radicals in type IIB fibers is two to three times higher than in type I fibers; this inherent difference causes a rapid decrease in type II fibers, especially type IIB, with age (Phu et al., 2015; Musumeci, 2017). Differences in mitochondrial function between type I and II fibers support a role for ROS in mitochondrial dysfunction (Harper et al., 2021).

### Effects of exercise on mitochondrial efficiency

High levels of ROS damage macromolecular structures, while low levels of ROS create greater resistance to stress and can even improve both longevity through adaptive defense responses and tolerance to subsequent stressors by activating mtDNA repair mechanisms (Corsetto et al., 2019). Acute production of exercise-induced ROS improves mitochondrial efficiency, while chronic increases in ROS are largely pathologic, indicating that exercise can increase the coupling efficiency of the ETC and reduce electron leakage and mitochondrial ROS overproduction, and restore homeostasis to optimal levels (Corsetto et al., 2019). Endurance training (ET) increases oxidative capacity, stimulates mitochondrial efficiency, and prevents age-related decreases in oxidative capacity. Endurance training in the elderly improves mtDNA integrity, mitochondrial density, SDH enzyme activity, ETC activity (such as complexes I, II, III, IV, and V) (Votion et al., 2010; Seo and Hwang, 2020; Harper et al., 2021). Moderate levels of exercise increase proteins of the ETC complex (III, IV, and V), the enzymatic activity of peroxisome proliferator-activated receptor-γ coactivator (PGC1-ɑ and PGC1-β), and TFAM gene expression in skeletal muscle (Parry et al., 2020). These findings support the notion that decreases in mitochondrial efficiency are likely the result of decreased activity levels, not due to the process of aging (Harper et al., 2021).

Muscle strength decreases faster than muscle mass does with age, which is primarily attributed to decreases in type II muscle fibers during sarcopenia. Resistance training (RT) increases the size and number of type II muscle fibers and is a good candidate for intervention against sarcopenia (Harper et al., 2021). In particular, RT increases the ratio of complexes IV/I + III, which in turn reduces electron leakage and ROS production from complexes I and III (the main sources of superoxide). In addition, RT in sedentary elderly increases ETC coupling by stimulating the activities of complex I, II, III, and IV, total MRC, NAD + synthesis, and mitochondrial respiration (Parise et al., 2005; Parry et al., 2020; Harper et al., 2021). These findings suggest that RT reduces oxidative stress by reducing ETC electron leakage and by increasing the ETC electron flux, rather than by regulating antioxidant activity. These results indicate that RT can, in some cases, stimulate mitochondrial function, increase the ratio of oxidative capacity to mitochondrial volume, and improve coupling efficiency (Harper et al., 2021). RT and ET have unique benefits in sarcopenia and aging, as simultaneous training improves ETC electron flux and mitochondrial coupling by increasing the expression and activity of complexes I, III, IV, and V (Balan et al., 2019; Harper et al., 2021). These exercises reduce oxidative damage to mtDNA, limits mtDNA mutations, preserves type II muscle fibers, and prevents sarcopenia. In addition, a combination of RT and ET increases the amount of mtDNA, stimulates mitochondrial protein synthesis, and increases mitochondrial biogenesis. A combination of strength-endurance (SE) training or endurance-strength (ES) training increases complexes I, II, III, IV, and V in ETC as well as CS and COX enzyme levels (Harper et al., 2021). The increase of complex II in the SE group is higher than that of the ES group, and SE is superior to ES because SE and ES stimulate mitochondria equally, while SE activates mTOR signaling in addition to stimulating mitochondria. These findings suggest that RT is preferred to ET in anti-aging exercise protocols (Harper et al., 2021).

### Effects of sarcopenia and exercise on antioxidant defenses

Oxidative stress is caused by an imbalance between the physiological production of free radicals and the potential of cells to neutralize them (Cesare et al., 2020). Age-related defects in MRC, together with superoxide generation by complex I, are important in the generation of oxidative stress (Guescini et al., 2017). Increases in the generation of ROS, reactive nitrogen species (RNS), and oxidative stress appear to be important causes of sarcopenia (Ji, 2002). Despite increased ROS production, aging appears to decrease the expression of antioxidant genes through increased lipid peroxidation, protein oxidation, and mtDNA damage, leading to mtDNA deletion, impaired redox signaling, increased apoptosis, and energy deficiency in conditions associated with sarcopenia (Ji, 2002). Age-related imbalances in ROS generation and antioxidant defense mechanisms are important causes of chronic inflammation in human skeletal muscle in sarcopenia. Redox signaling also alters enzymatic activity, induction of transcription factors, DNA binding, and gene expression (Guescini et al., 2017). Mammalian cells activate several signaling pathways following oxidative stress, including kappa B nuclear factor (NF-κB), PGC1-ɑ, mitogen-activated protein kinase (MAPK), phosphoinositide 3-kinases (PI3Ks)/protein kinase B (PKB, also known as Akt and P53 pathways) (Ji, 2002; Guescini et al., 2017). These pathways are also activated by exercise which increases H_2_O_2_ levels (Guescini et al., 2017). For example, $O_{2}^{-}$ /H_2_O_2_ formed in the mitochondrial matrix activates mitogen-activated protein p38 kinase (p38 MAPK). In addition, NF-κB-responsive elements are present in the promoter regions of genes encoding antioxidants such as catalase (CAT), glutathione peroxidase (GPx), and Mn- and Cu-Zn-superoxide dismutase (SOD) (Guescini et al., 2017). These antioxidants stabilize ROS levels by eliminating free radicals, regulating ROS/RNS-producing enzymes, and/or through other adaptive mechanisms (Cesare et al., 2020). In addition, muscle fibers have antioxidant defense mechanisms that reduce the risk of oxidative damage. Hence, enzymatic and non-enzymatic (i.e., vitamin A, vitamin C, vitamin E, β-carotene) antioxidant systems regulate the redox status of myocytes. However, antioxidants such as CAT, SOD, peroxiredoxins (PRDXs), GPx, and glutathione transferase (GSH) are enzymatic antioxidants that are reduced in muscles during aging (Cesare et al., 2020).

Catalase (CAT), a high molecular weight tetrameric enzyme, reduces oxidative stress by degrading cellular H_2_O_2_ to produce water (H_2_0) and oxygen (Cesare et al., 2020). Levels of catalase mRNA expression in muscle decrease with age (Lambertucci et al., 2007; Cho et al., 2008), and increases in H_2_O_2_ and decreases in CAT levels are associated with sarcopenic aging (Sánchez‐Castellano et al., 2020). Hence, appears that H_2_O_2_ is a major cause of sarcopenia and that decreases in CAT and GPx indicate that antioxidant dysfunction may mark the onset of sarcopenia (Sánchez‐Castellano et al., 2020). There is evidence that lower levels of CAT characterize the primary inflammatory response during sarcopenia (Cruz-Jentoft et al., 2021). In contrast, delivering CAT to mitochondria increases CAT activity by 2 to 10-fold in skeletal and cardiac muscle. Targeted expression of CAT in mitochondria increases cardiac function, athletic performance, and longevity (20%), and counteracts oxidative muscle damage (Li et al., 2009). Increased expression of mitochondrial CAT in muscle prevents muscle atrophy in a mouse model of sarcopenia (Li et al., 2009). Mice with augmented muscle mitochondrial CAT expression have altered mitochondrial function such as decreased ROS production and increased maximum life spans (Xu et al., 2021) and attenuated dystrophin deficiency and muscle atrophy (Selsby, 2011). Levels of CAT activity are reduced in sedentary rats, while exercise increases CAT activity and CAT mRNA expression (Cho et al., 2008; Teixeira et al., 2012). Moderate physical activity such as treadmill and aerobic training increases antioxidant enzymes such as CAT, SOD, and GPx in animals and humans (De Castro et al., 2009; Vilela et al., 2018; Parry et al., 2020; Matta et al., 2021). Two hours of training also increased CAT enzyme activity in both type 1 and 2 muscle fibers in rats (Mendes et al., 2021). Increased CAT expression inhibits mitochondrial dysfunction, maintains muscle mass and energy production, improves exercise capacity, and prevents reductions in muscle fiber diameter, suggesting that expression of muscular mitochondrial CAT can prevent sarcopenia-related phenotypes (Xu et al., 2021).

Mammalian cells express three coding genes for SOD, an antioxidant enzyme that uses copper/zinc or manganese ions as active sites (Cesare et al., 2020). The abundance of mRNA for Cu/ZnSOD, MnSOD, and GPX in muscle types remains unchanged or decreases with increasing age (Ji, 2002). Although SOD1 is present both in the cytosol and in the mitochondrial intermembrane space, SOD2 is located in the mitochondrial matrix (Mason et al., 2020). Cytoplasmic and mitochondrial SOD (Cu/ZnSOD and MnSOD, respectively) convert $O_{2}^{-}$ to oxygen and H_2_O_2_, which is subsequently converted to water by CAT or GPx (Cesare et al., 2020). A lack of Cu/Zn SOD is associated with aging, premature muscle atrophy and weakness (sarcopenia), increased mitochondrial H_2_O_2_, stunted muscle growth, decreased muscle mass, decreased lifespan, increased mitochondrial ROS, and increased oxidative damage (Homma et al., 2018; Corsetto et al., 2019; Kadoguchi et al., 2020; Sataranatarajan et al., 2020). However, exercise-induced increases in CAT and SOD coincide with elevations in H_2_O_2_ and $O_{2}^{-}$ levels, respectively (Pascual-Fernández et al., 2020). In addition, endurance and resistance exercise also increase SOD activity, MnSOD protein levels, mRNA abundance for MnSOD, SOD2, and CAT activity (Ji, 2002; Lambertucci et al., 2007; Shahar et al., 2013; Ceci et al., 2020; Cesare et al., 2020; Mason et al., 2020; Serra et al., 2020). On the other hand, levels of Mn-SOD protein negatively correlate with caspase-3 (a marker of apoptosis), while positively correlating with BCL-2 and HSP70 (anti-apoptosis markers) (Siu et al., 2004). These studies suggest the positive effects of exercise on the expression of antioxidants to counter the effects of sarcopenia.

Peroxiredoxins (PRDXs) are a family of antioxidant enzymes that reduce hydroperoxides to water in the presence of electron donors (Jackson, 2016). There are six types of peroxiredoxin antioxidant enzymes in mammals that are located in the cytosol (PRDX1, PRDX2, PRDX6), mitochondria (PRDX3, PRDX5), and endoplasmic reticulum (PRDX4) (Gruber et al., 2015). PRDXs are responsible for disposing of 90% of ubiquitous cellular peroxides, and act as local H_2_O_2_ regulators (Galli et al., 2021). PRDX3-null mice have reduced physical strength and mtDNA levels compared to wild ten-month-old mice, indicating that PRDX3 deficiency increases oxidative stress, mitochondrial dysfunction, and aging (Galli et al., 2021). Suppression of mitochondrial PRDX leads to decreases in ATP levels, reduced strength, lowered mtDNA content, and shortened lifespans, while it increases mitochondrial uncoupling, ROS levels, and oxidative damage of DNA and proteins (Lee et al., 2014; Gruber et al., 2015). In addition, the absence of PRDX3 increases peroxide concentrations and diminishes regulation of the mitochondrial membrane potential, causing further muscle fatigue in mice (Wadley et al., 2016). Levels of mitofusin 1 and 2 protein levels decrease in muscles of older PRDX3-deficient mice, likely due to abnormalities in mitochondrial fusion (Lee et al., 2014). In addition, muscle atrophy occurs in PRDX6−/−mice with an increase in muscle RING-finger protein-1 (MuRF1) levels, suggesting that PRDX6 deficiency causes premature aging as deletion of PRDX6 creates a sarcopenic phenotype *via* the IGF-1/Akt-1/FOXO1 pathway, coupled with reduced muscle differentiation and protein synthesis (Pacifici et al., 2020). The removal of both SOD1 and PRDX4 increases the sensitivity of muscle to ROS that exacerbates muscle damage, augments oxidative stress and lipid peroxidation, leading to the release of cytochrome c, which then disrupts ETC function and increases apoptosis (Homma et al., 2018). In contrast, overexpression of PRDX3 leads to repair of sarcopenic atrophy, decreased ROS production, increased oxygen consumption in mitochondrial complex II (Ahn et al., 2018), and improved contractile force by muscles (Van Remmen et al., 2019). In addition, high-intensity exercise and moderate exercise increases the expression of PRDX1, PRDX3, PRDX4, PRDX5, CuZnSOD, SOD2, and SOD3 (Brinkmann et al., 2012; Trewin et al., 2018; Brown et al., 2019; Mason et al., 2020; Borges et al., 2021; Galli et al., 2021). Increased PRDX expression levels following exercise suggests an important role of PRDX in cell regeneration, likely *via* NF-κB oxidation-sensitive transcription factors (Wadley et al., 2016). In summary, PRDXs modulate mitochondrial production of ROS induced by exercise (Lightfoot and Cooper, 2016), as shown by the adaptive role of PRDX3 in modulating H_2_O_2_ in response to exercise training (Lightfoot and Cooper, 2016; Wadley et al., 2016). However, mitochondrial ROS neutralization alone is not sufficient to inhibit reduced skeletal muscle mass and contractile function with age (Eshima et al., 2020), as exercise creates mild oxidative stress, which in turn stimulates the expression of antioxidant enzymes (Ji, 2002).

### Effects of sarcopenia and exercise on mitochondrial dynamics

Mitochondria produce ATP and are essential for the regulation of various cell functions (Memme et al., 2021), also undergo fission and fusion to maintain a healthy mitochondrial network (Romanello, 2021). Mitochondrial fission isolates dysfunctional mitochondria from healthy mitochondria, while mitochondrial fusion reduces mitochondrial dysfunction by increasing network coupling and facilitating the redistribution of metabolites, proteins, and mtDNA (Yoo et al., 2018). The balance between mitochondrial fusion and fission is altered in sarcopenic muscles. Impaired mitochondrial fission leads to reduced mitophagy, leading to an accumulation of dysfunctional organelles (Romanello, 2021). Mitochondrial fission requires the joint action of proteins such as dynamin-related protein 1 (DRP1), fission protein 1 (FIS1), and mitochondrial fission factor (MFF) (Memme et al., 2021). Decreases in DRP1 activity in sarcopenia lead to muscle atrophy, systemic metabolic disorders, abnormal mitochondrial function, and disorders of autophagy and mitophagy (Romanello, 2021). Expression levels of FIS1 also decrease in old age (Picca et al., 2017), and older animals have lower levels of FIS1 and autophagy than younger animals, resulting in impaired autophagy in older muscles (Joseph et al., 2013), while knockdown of FIS1 activates muscle atrophy in sarcopenia (Calvani et al., 2013). Hence, reductions of mitochondrial fission proteins (such as DRP1, MFF, and FIS1) in patients with sarcopenia inhibit mitophagy and leads to the accumulation of dysfunctional organs and muscle atrophy (Chen et al., 2018). In contrast, removal of the MFF inhibitor in aging muscle leads to increased mitochondrial fission and mitophagy and improves mitochondrial function and longevity (Romanello, 2021). In addition, exercise appears to facilitate the removal of damaged mitochondria, improve the formation of new mitochondria, and increase the capacity of the mitochondrial network (Chen et al., 2018). Hence, levels of DRP1 protein are higher in trained old mice than in young mice (Gusdon et al., 2017). Exercise in elderly patients increases DRP1 phosphorylation at ser637, indicating the presence of highly efficient mitochondria (Casuso and Huertas, 2020). These findings suggest that mitochondrial fission proteins such as DRP1, MFF, and FIS1 are likely to be reduced in people with sarcopenia, while exercise increases mitochondrial fission markers including DRP1, FIS1, and MFF (Hoffman et al., 2015; Yoo et al., 2018; Guan et al., 2019; Di Liu et al., 2021).

Fusion elongates the mitochondrial network and is mediated by proteins such as mitofusin 1 (Mfn1), mitofusin 2 (Mfn2), and optic atrophy (OPA1). The simultaneous ablation of Mfn1 and Mfn2 in skeletal muscle in mice leads to mitochondrial dysfunction, mitochondrial damage, muscle atrophy, growth retardation, and premature death. In particular, deletion of Mfn2 in young animals causes muscle atrophy due to extensive mitochondrial fragmentation, ROS production, endoplasmic reticulum stress, inhibition of autophagy, and exacerbation of sarcopenia (Harper et al., 2021; Romanello, 2021). Expression levels of Mfn2, Fis1, Drp1, and Opa1 in sarcopenic muscle are down-regulated, suggesting a role for mitochondrial dysfunction in the pathogenesis of sarcopenia and skeletal muscle atrophy (Di Liu et al., 2021). Deletion and inhibition of OPA1 also causes mitochondrial dysfunction, increased levels of IL6, IL1, ROS, mitochondrial DNA damage, and subsequent stimulation of transcription factors such as FoxO3 and NF-κB which are associated with sarcopenia and premature death (Romanello, 2021). In addition, suppression of Mfn2 and OPA1 in skeletal muscles of elderly mice exacerbates metabolic changes and sarcopenia due to impaired mitochondrial quality and autophagy (Chang et al., 2019). A deficiency of OPA1 can disrupt mitochondrial fusion during biogenesis and cause defective mitochondria (Caffin et al., 2013). Levels of the mitochondrial fusion protein OPA1 decrease with age and inactivity (Romanello, 2021). However, overexpression of OPA1 has a protective effect on myopathy (Romanello, 2021). In contrast, acute bouts of endurance exercise increase mRNA levels of Mfn1, Mfn2, and Drp1 in humans and rats, and elevations in Mfn1 and Mfn2 protein levels persist after long-term resistance training or endurance exercise in rat skeletal muscles (Chen et al., 2018; Liu et al., 2021). The increased ratio of Mfn2/Drp1 in older mice is attenuated by exercise (Liu et al., 2021). Endurance exercise increases OPA1 gene expression in insulin-resistant obese elderly people (Chen et al., 2018), while aerobic exercise increases OPA1 mRNA content in the skeletal muscle of the elderly (Moreira et al., 2017). In summary, the balance between fusion and fission required for mitochondrial function in skeletal muscle is often compromised during aging. Exercise increases the ratio of fusion to fission proteins in the elderly, allowing the formation of tubular mitochondrial networks as an adaptation to changes in the cellular environment (Harper et al., 2021; Liang et al., 2021).

### Roles of autophagy and mitophagy

**FIGURE 2:**
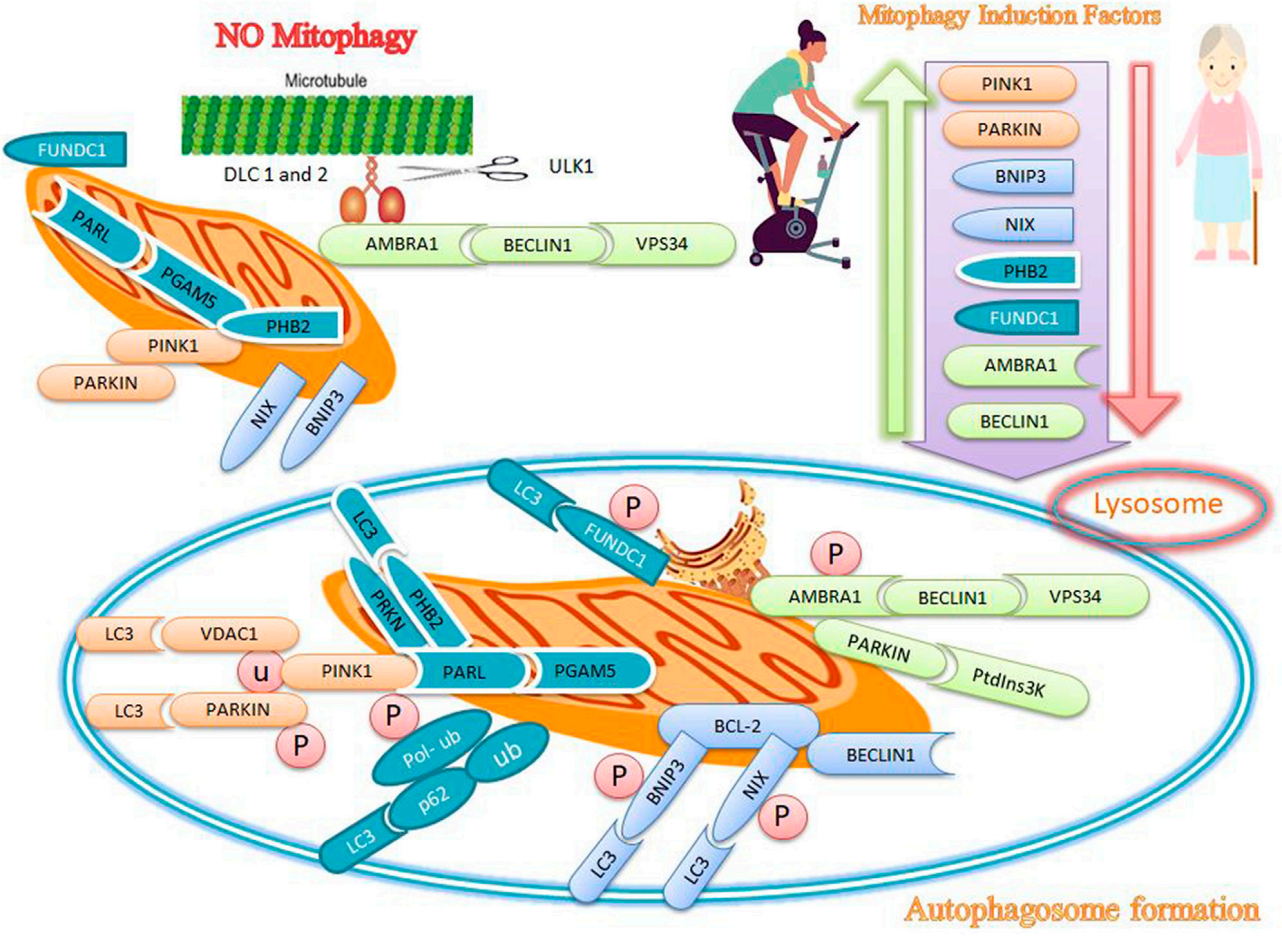
Effects of exercise and sarcopenia on mitophagy markers. Sarcopenia decreases mitophagy markers, while exercise appears to increase mitophagy markers such as PINK1, PARKIN, BNIP3, NIX, PHB2, FUNDC1, AMBRA1, and BECLIN1.

Autophagy is a programmed and genetic catabolic process that removes cellular proteins and excessive organelles through the formation of autophagosomes, which combines with lysosomes to form autolysosomes where the enveloped contents are destroyed. Autophagosomes mediate non-selective or selective autophagy. Non-selective autophagy, which is activated in response to starvation or nutrient deprivation, supplies essential amino acids and nutrients for cell survival. In contrast, selective autophagy describes the specific removal of damaged or excessive organelles; mitophagy is a form of selective autophagy for the removal of damaged or dysfunctional mitochondria (Ding and Yin, 2012) to maintain mitochondrial quality under both physiological and conditions of cellular stress (Romanello, 2021). The regulation of autophagy and mitophagy maintains muscle mass, while excessive autophagy/reduced mitophagy contributes to muscle atrophy (Romanello, 2021). When damaged mitochondria are not removed, apoptosis signaling is activated to destroy the nucleus, which simultaneously or independently activates the autophagy pathway and ubiquitin ligase (that mediates protein degradation). In addition, necrosis is initiated to remove muscle proteins, mitochondria, and nuclei in dysfunctional mitochondria (Alway et al., 2017). Mitophagy in mammals is regulated by PTEN-induced kinase 1 (PINK1), parkin, Bcl2/adenovirus E1B 19 kDa protein-interacting protein 3 (BNI3), and NIP3-like-protein X (NIX), prohibitin 2 (PHB2), FUN14 domain containing 1 (FUNDC1), activating molecule in BECN1-regulated autophagy protein 1) AMBRA1 (Figure 2).

### Effects of sarcopenia and exercise on PINK1 and parkin mitophagy markers

PINK1 and parkin are attractive candidates for targeting dysfunctional mitochondria as the accumulation of PINK1 on the outer mitochondria membrane (OMM) provides an important mechanism for identifying damaged mitochondria (Alway et al., 2017). When the membrane potential of damaged mitochondria is depolarized, the entry of PINK1 into the inner mitochondria membrane (IMM) is inhibited, allowing the protein to accumulate on the OMM and absorb parkin from the cytosol to the OMM. Parkin is a ubiquitin ligase E3 and its activity is induced by PINK1-dependent phosphorylation (Di Liu et al., 2021) (Figure 2). Activated parkin then ubiquitinates OMM proteins such as voltage-dependent anion channel 1 (VDAC1) and produces ubiquitin (Ub) and poly-ubiquitin (poly-Ub) chains. Poly-Ub chains are subsequently phosphorylated by PINK1 and mediates autophagy. Ubiquitin-binding adapter proteins, such as p62, optineurin (OPTN), and nuclear dot protein 52 (NDP52), identify phosphorylated poly-Ub chains on mitochondrial proteins and absorb damaged mitochondria through interaction with microtubule-associated protein light chain 3 (LC3) (Di Liu et al., 2021) (Figure 2). Parkin connects simultaneously to LC3 in the newly formed phagophore (Moreira et al., 2017). Deletion of parkin reduces muscle mass and leads to poor physical function in older mice, while overexpression of parkin improves skeletal muscle function in older mice (Di Liu et al., 2021). Parkin causes proteasomal degradation of mitofusins, favoring increased fission and suppressing mitochondrial fusion, and allows for the separation of defective mitochondria from healthy mitochondrial networks (Di Liu et al., 2021). PINK1 can indirectly activate Drp1 to promote the degradation of defective mitochondria (Di Liu et al., 2021). The absence of PINK1 or parkin leads to the accumulation of dysfunctional and fragmented mitochondria, indicating that PINK1 and parkin may be an important targets that regulate damaged mitochondria (Drake et al., 2016). Similarly, decreased bioavailability of PINK1 and parkin1 may indicate mitochondrial dysfunction in old age (Moreira et al., 2017). Genetic deletion of PINK1 and parkin leads to dysfunction of mitophagy and mitochondria, muscle destruction, and reduced longevity, while increased mitophagy with overexpression of PINK1, parkin, and DRP1 in *drosophila* reduces age-related muscle dysfunction and increases longevity (Romanello, 2021). Exercise increases PINK1 and parkin mRNA expression in skeletal muscle of mice (Drake et al., 2016; Zhao et al., 2018a), and improves mitochondrial function through mitochondrial fusion/fission signaling (Zhao et al., 2018b). In addition, exercise combined with dietary restriction leads to mitochondrial abnormalities and myofibrillar injuries, with increased levels of PINK1 and Drp1 likely related to the activation of mitophagy (Zhao et al., 2018a). Since parkin and PINK1 regulate mitophagy and mediate sarcopenia-related muscle weakness, exercise-induced increases in the expression of these proteins may likely be a useful strategy to form a healthy mitochondrial network in skeletal muscles Table 1.

**TABLE 1:** A summary of exercise studies on the factors affecting autophagy and mitophagy.

Type of exercise	Intensity and duration	Autophagy and mitophagy factors	Changes	References
Swimming training	10 weeks, 5 days/week, the first week 20 min every day and then increased 10 min per week. The final 3 weeks’ duration was at 90 min/per day	PINK1, parkin and Drp1	Increased	Drake et al. (2016); Zhao et al. (2018a); Zhao et al. (2018b)
Short-term intense exercise	One-hour treadmill	LC3-II	Increased	Grumati et al. (2011)
Cycling	≥6 h training a week for at least 5 years	BNIP3 and parkin	Increased	Jamart et al. (2013); Balan et al. (2019)
6-min walk	at least 90 min of moderate activity a week over the last year	OPA1, PINK1, parkin, BNIP3, and NIX	Increased	(Drummond et al. (2014); Moreira et al. (2017); Zhao et al. (2018a)
Chronic high intensity swimming training	8 weeks of swimming training, 7 days/week	Prohibitin	Increased	Sun et al. (2008)
Chronic aerobic training	12 weeks, 60 min/day, belt speed (8–20 m/min)	Prohibitin, ATP formation, mtDNA content, and complex I, II, and III activity	Increased	Gu et al. (2014a)
Moderate-intensity training	Treadmill training was performed at a slope of 10°, running at 10–15 m/min, 10–60 min per day, 6 days per week for 8 weeks	Prohibitin, complex V	Increased	Fang et al. (2020)
High-intensity exercise	25-min run on a treadmill	Mice lacking FUNDC1	Decreased time and distance	Fu et al. (2018)
Acute treadmill running	a single bout (90 min) of treadmill running	Ulk1	Increased	Laker et al. (2017)
Endurance training and voluntary physical activity	12-week treadmill and 12-week voluntary free wheel	PGC-1α and mtTFA, Beclin-1, LC3-II, LC3II/LC3I, and Parkin	Increased	Santos-Alves et al. (2015)
Acute aerobic exercise	swimming for 10 min during 3 consecutive days	Ulk1, LC3-II	Increased	Lenhare et al. (2017)
Running training on treadmill	with a 5-degree incline at a speed of 16.4 m/min for 40 min/day, 5 days a week for 8 weeks	Beclin-1, ATG7, AMBRA1	Increased	Kim et al. (2013); Rędowicz, (2021)

### Effects of sarcopenia and exercise on BNIP3 and NIX mitophagy markers

BNIP3 and NIX are mitophagy receptors on mitochondria that bind to LC3 and link mitochondria to the autophagosome (Romanello, 2021). BNIP3 and NIX are located on the mitochondrial surface, and both can cause mitochondrial depolarization that leads to mitophagy (Zhang and Ney, 2009). BNIP3 and NIX also increase the amount of Drp1 in mitochondria during the early stages of mitophagy, which causes parkin cleavage and recruitment (Triolo and Hood, 2021). On the other hand, BNIP3 and NIX bind to Bcl-2 and resolve the beclin1/Bcl-2 interaction, so that beclin1 can initiate mitophagy. In addition, BNIP3 and NIX are phosphorylated to form homodimers on the OMM and then bind to LC3 (Moreira et al., 2017). The quantitative and qualitative maintenance of mitochondria depends on maintaining adequate concentrations of several proteins such as BNIP3, NIX, PGC-1α, TFAM, OPA1, Drp1, Mfn1, Mfn2, PINK1, and parkin, which are involved in the mitochondrial biogenesis, mitochondrial dynamics and mitophagy process. There are changes in the concentration and function of these proteins during aging (Moreira et al., 2017). Genetic deletion of NIX in mice leads to pathological cardiac hypertrophy and reduced contractility with age; on the other hand, lack of NIX and BNIP3 causes age-related mitochondrial cardiomyopathy (Dorn, 2010; Zhao et al., 2018a).

The expression of some mitophagy regulators such as LC3, BNIP3, beclin1, Atg7, p62, and parkin decreases with age, indicating that sarcopenia is associated with dysfunctional or deficient mitophagy (Zhao et al., 2018a; Yoo et al., 2018; Liang et al., 2020). In contrast, intense endurance training increases autophagy in skeletal muscle, as shown by the increased conversion of LC3-I to LC3-II (Grumati et al., 2011; Ogura et al., 2011; Liang et al., 2020). Aerobic exercise increases mitophagy, accelerates the removal of metabolic wastes and damaged proteins in cells, and ultimately improves the functional and structural regeneration of the skeletal system (Liang et al., 2021). In addition, mitophagy receptors such as BNIP3 or NIX can bind directly to LC3 to absorb autophagosomes and facilitate mitochondrial removal (Di Liu et al., 2021). In this regard, endurance training increases the expression of BNIP3 and parkin in the skeletal muscles of the elderly and eliminates oxidative damage and damaged mitochondria by autophagy-lysosomal interactions (Zhang and Ney, 2009; Jamart et al., 2013; Balan et al., 2019). Exercise increases BNIP3 and NIX expression in skeletal muscle, where this targets damaged mitochondria for degradation through mitophagy (Drake et al., 2016). Skeletal muscle contraction during exercise improves mitochondrial quality by upregulating OPA1, PINK1, parkin, BNIP3, and NIX (Drummond et al., 2014; Moreira et al., 2017). Slow-twitch muscle has more mitophagy flux than fast-twitch muscle due to increased levels of BNIP3 (Zhao et al., 2018a); increased mitophagy and BNIP3 levels can protect cells against ROS due to hypoxia or exercise (Zhao et al., 2018a).

### Effects of sarcopenia and exercise on prohibitin

Prohibitins are located in the IMM and are receptors for parkin-mediated mitophagy in mammalian cells (Strappazzon et al., 2020). Prohibitin 1 and 2 (PHB1 and PHB2) assemble in a ring-like prohibitin complex at the IMM. PHB2 enhances PINK1-PRKN-mediated mitophagy by stabilizing PINK1 and increasing PRKN recruitment to mitochondria. PHB2 regulates the PINK1-PRKN pathway, but negatively regulates PARL (presenilin-associated rhomboid-like) activity (Yan et al., 2020). Moreover, the PARL-PGAM5 axis is required for the PHB2-mediated PINK1 stabilization in depolarized mitochondria, because PHB2 binds to PGAM5 to protect against processing by PARL. In contrast, PHB2 tends to bind to PARL after mitochondrial depolarization, and the release of PGAM5 retains PINK1 on the OMM and subsequently initiates mitophagy. PINK1 recruits PRKN to the mitochondria and then allows for ubiquitination and degradation of some OMM proteins, leading to mitophagy (Yan et al., 2020) (Figure 2). Rupture of the OMM rupture makes PHB2 to interact with LC3 in the IMM (Signorile et al., 2019). The accumulation of unfolded proteins in the mitochondrial matrix also stimulates the uptake of PRKN into the mitochondria (in addition to changes in the mitochondrial membrane potential) (Yan et al., 2020). PHB1 and PHB2 stimulate a wide range of cellular functions, including mitophagy, cellular signaling, mitochondrial biogenesis, aging, and apoptosis (Blottner et al., 2021).

Overexpression of PHB2 increases PRKN-dependent mitophagy (Yan et al., 2020). Mitophagy mediated by PHB1 and PHB2 is protective improves longevity by maintaining mitochondrial function, while a deficiency of PHBs shortens the lifespan (Blottner et al., 2021). PHB proteins are expressed in high-energy cells, which are more prone to mitochondrial dysfunction because PHBs complexes stimulate the ATP synthase complex. The upregulation of PHB1 increases complex I activity. However, PHB2 acts as a chaperone for the stabilization of the subunits of the mitochondrial respiratory complex. Silencing of the PHB complex reduces the activity of other complexes in the respiratory chain, and dysfunction of PHBs proteins is associated with aging. Reduced expression of PHB2 decreases the activity of mitochondrial HCLS1-associated protein X-1 (HAX1), an anti-apoptotic protein, which reduces mitochondrial integrity and increases in caspase 9/3 activity and apoptosis (Signorile et al., 2019). Hence, decreased PHB impairs mitochondrial biogenesis and structural integrity, leading to a decrease in complex I efficiency and an increase in ROS formation (López-Lluch, 2017). Parkin-mediated mitochondrial damage increases the binding of the PHB2 complex to LC3-II protein, indicating that PHB2 is required for mitochondrial elimination and may contribute to its role in aging (Wei et al., 2017).

Overexpression of PHB1 increases the number of copies of mtDNA and several mitochondrial proteins such as PGC-1α, complex IV, NRF2, OPA1, DRP1, and TFAM (Signorile et al., 2019). Exercise increases PHB1 levels and attenuates age-related mitochondrial dysfunction. For example, swimming for 8 weeks increases PHB1, PHB2, malate dehydrogenase, triosephosphate isomerase, ATP synthase (complex IV), and isocitrate dehydrogenase (Sun et al., 2008; Hussey et al., 2013). Chronic aerobic exercise increases PHB, ATP formation, mtDNA content, and complex I, II, and III activity in older mice while reducing ROS and mitochondrial swelling (Gu et al., 2014a). Increases in PHB1 expression after 8 weeks of moderate-intensity exercise positively correlates with ATP content and V-complex activity and negatively with ROS levels (Fang et al., 2020). Thus, PHBs-mediated mitophagy reduces age-related mitochondrial dysfunction while increasing the coupling of energy-producing complexes, suggesting that physical activity can be used to increase the quality of life during sarcopenia.

### Effects of sarcopenia and exercise on FUNDC1

FUNDC1 is a protein located on the OMM and acts as a mitophagy receptor that regulates mitochondrial removal in mammals by binding to LC3 under hypoxic conditions (Figure 2). FUNDC1 is the molecular link that integrates mitochondrial fission and mitophagy at the interface of the endoplasmic reticulum (ER)–mitochondrial contact site (MAM). For example, the FUNDC1 mitophagy receptor is dephosphorylated under hypoxic conditions to increase its interaction with LC3 and induce mitophagy (Gan et al., 2018). FUNDC1 can also participate in mitophagy by activating the AMPK-ULK1 pathway. Increases in AMPK expression stimulate autophagy-related FUNDC1 and LC3 protein levels (Gao et al., 2020) (Figure 2)*.* Exercise alters energy metabolism by increasing AMP levels and decreasing ATP levels, and activating the AMPK energy sensor. AMPK phosphorylates ULK1 to induce autophagy under hypoxic conditions (Gao et al., 2020). ULK1 is upregulated and absorbs fragmented mitochondria under hypoxic conditions. In addition, phosphorylation of p-ULK1 correlates with increased levels of FUNDC1, suggesting an interaction of these two pathways in mitophagy (Gao et al., 2020).

The loss of FUNDC1 in muscle reduces mitochondrial energy and athletic performance and leads to defects in mitophagy (Fu et al., 2018; Gan et al., 2018) and reduced exercise capacity (Fu et al., 2018; Triolo and Hood, 2021). Aging is associated with an accumulation of mtDNA damage and disruption of the mitophagy program induced by BNIP3L, FUNDC1, and NIX (Hepple, 2014; Lampert et al., 2019), suggesting that these factors do not mediate the removal of damaged mitochondria and do not improve the effects of aging (Lampert et al., 2019). A study of 18- to 20-month-old mice reported that one session of treadmill running and 9 weeks of resistance training activated AMPK/Ulk1-dependent autophagy in skeletal muscle, and reduced myocyte apoptosis (Laker et al., 2017; Harper et al., 2021). FUNDC1 is also involved in fat utilization, increased exercise capacity, mitochondrial quality control, and maintenance of metabolic homeostasis (Fu et al., 2018).

Irisin, a myokine secreted during exercise, increases FUNDC1 and mitophagy. Irisin reduces oxidative stress, mitochondrial dysfunction, and apoptosis through FUNDC1-dependent mitophagy while increasing ATP generation. However, the beneficial effects of irisin on complex I/III activities are eliminated by silencing FUNDC1. Thus, FUNDC1-associated mitophagy may be a protective pathway involving the destruction of damaged mitochondria by lysosomes. Irisin also increases antioxidant capacity by stimulating GSH, SOD, and GPX, but these benefits are eliminated after silencing FUNDC1. In addition, the activation of caspase-3 and apoptosis by irisin is reduced by FUNDC1, while the removal of FUNDC1 increases the activation of caspase-3 and apoptosis (Jiang et al., 2021). Thus, exercise-induced secretion of irisin has several roles in FUNDC1-mediated mitophagy, including degradation of injured mitochondria, increased ATP production, increased complex I/III, reduced oxidative stress, increased antioxidant capacity, reduced caspase-3 activity, and reduced mitochondria-dependent apoptosis. While these changes are negatively regulated in sarcopenia. Hence, there is strong evidence that exercise can be a non-pharmacological intervention to increase mitophagy in people with sarcopenia.

### Effects of sarcopenia and exercise on AMBRA1

AMBRA1 is a mitophagy receptor able to remove inefficient mitochondria in mammalian cells (Strappazzon et al., 2020). AMBRA1 and FUNDC1 are OMM proteins that can identify damaged mitochondria and regulate mitophagy (Yao et al., 2021). AMBRA1 prevents Beclin1-dependent autophagy (Gu et al., 2014b), and improves Beclin 1 interaction with its target, VPS34, thus regulating autophagosome formation (Di Bartolomeo et al., 2010). The AMBRA1–Beclin1–Vps34 complex is bound to the dynein motor complex *via* a specific interaction with dynein light chains (DLCs) 1 and 2 in the absence of autophagy. ULK1 phosphorylates AMBRA1 when autophagy is activated, and then releases AMBRA1 from the dynein complex (Schoenherr et al., 2020) (Figure 2)*.* The ULK1-mediated phosphorylation of AMBRA1 drives its translocation to the endoplasmic reticulum (ER) and activates autophagy. These findings indicate an important role for AMBRA1 in the membrane scrambling activity of organelles (mitochondria and endoplasmic reticulum) leading to autophagosome formation (Manganelli et al., 2020) (Figure 2).

AMBRA1 interacts with parkin after translocation to depolarized mitochondria. Prolonged mitochondrial depolarization enhances the interaction of AMBRA1 and parkin, where it activates the PtdIns3K complex of damaged mitochondria and participates in autophagy (Fimia et al., 2013). Deletion of AMBRA1 is not as effective in transporting parkin to depolarized mitochondria but inhibits the deletion of damaged mitochondria. Conversely, overexpression of AMBRA1 enhances the removal of depolarized mitochondria by interacting with parkin (Fimia et al., 2013). DRP1 and parkin are not as well absorbed in the mitochondria, and are associated with lysosomal abnormalities, abnormal mitochondrial accumulation, and decreased I-III complex activity (Rędowicz, 2021). On the other hand, levels of AMBRA1 decrease in gastrocnemius muscles of older mice (Lenhare et al., 2017), and functional deficiency of AMBRA1 in mice causes growth retardation, autophagy dysfunction, accumulation of ubiquitin proteins, unbalanced cell proliferation, and increased apoptosis (Fimia et al., 2007; Fimia et al., 2013).

Exercise increases autophagy-related proteins such as Beclin-1 and parkin that are associated with AMBRA1, and these factors increase mitochondrial density caused by endurance training and physical activity (Santos-Alves et al., 2015). Levels of Beclin-1, AMBRA1, autophagosome formation, and autophagy reduce in aging but recover by exercise (Kim et al., 2013; Lenhare et al., 2017). In addition, key autophagy regulators such as Beclin-1, p62, LC3-II, and Ulk1 in the gastrocnemius muscle of 24-month-old mice increase after 8 weeks of running on a treadmill (Harper et al., 2021). In general, decreased levels of mitophagy markers such as PINK1, parkin BNIP3, NIX, PHB2, FUNDC1, and AMBRA1 are associated with a reduced quality of life in sarcopenia associated with aging. Thus exercise improves mitochondrial dynamics, increases mitochondrial biogenesis, and maintains a healthy mitochondrial network, leading to exercise induced improvements in mitochondrial quality by stimulating mitophagy.

### Effects of sarcopenia and exercise on mitochondrial biogenesis

Mitochondrial biogenesis allows for the efficient transcription, translation, and importation of new proteins into existing organs in response to stimuli such as exercise, muscle contractions, and aging (Romanello, 2021). Sarcopenia is associated with a decrease in mitochondrial biogenesis. Nuclear and mitochondrial genomes regulate the expression of transcription factors for mitochondrial biogenesis, the most important of which is PGC-1α (Dozio et al., 2021). PGC-1α and its family members [PGC-1β and PGC-1-associated activator (PRC)] initiate gene transcription by binding to transcription factors (Memme et al., 2021). The coactivators PGC-1α and PGC-1β, which are the master regulators of mitochondrial biogenesis, are activated by stimuli that alter cellular energy demands such as exercise, fasting, and cold exposure. PGC-1α and PGC-1β, which lack DNA binding domains, activate transcription factors such as transcriptional factor A mitochondrial (TFAM), myocyte enhancing factors (MEFs), nuclear respiratory factors (NRFs), estrogen-related receptor (ERR), forkhead box (FoxOs) and peroxisome proliferator-activated receptors (PPARs) (Romanello, 2021).

**FIGURE 3:**
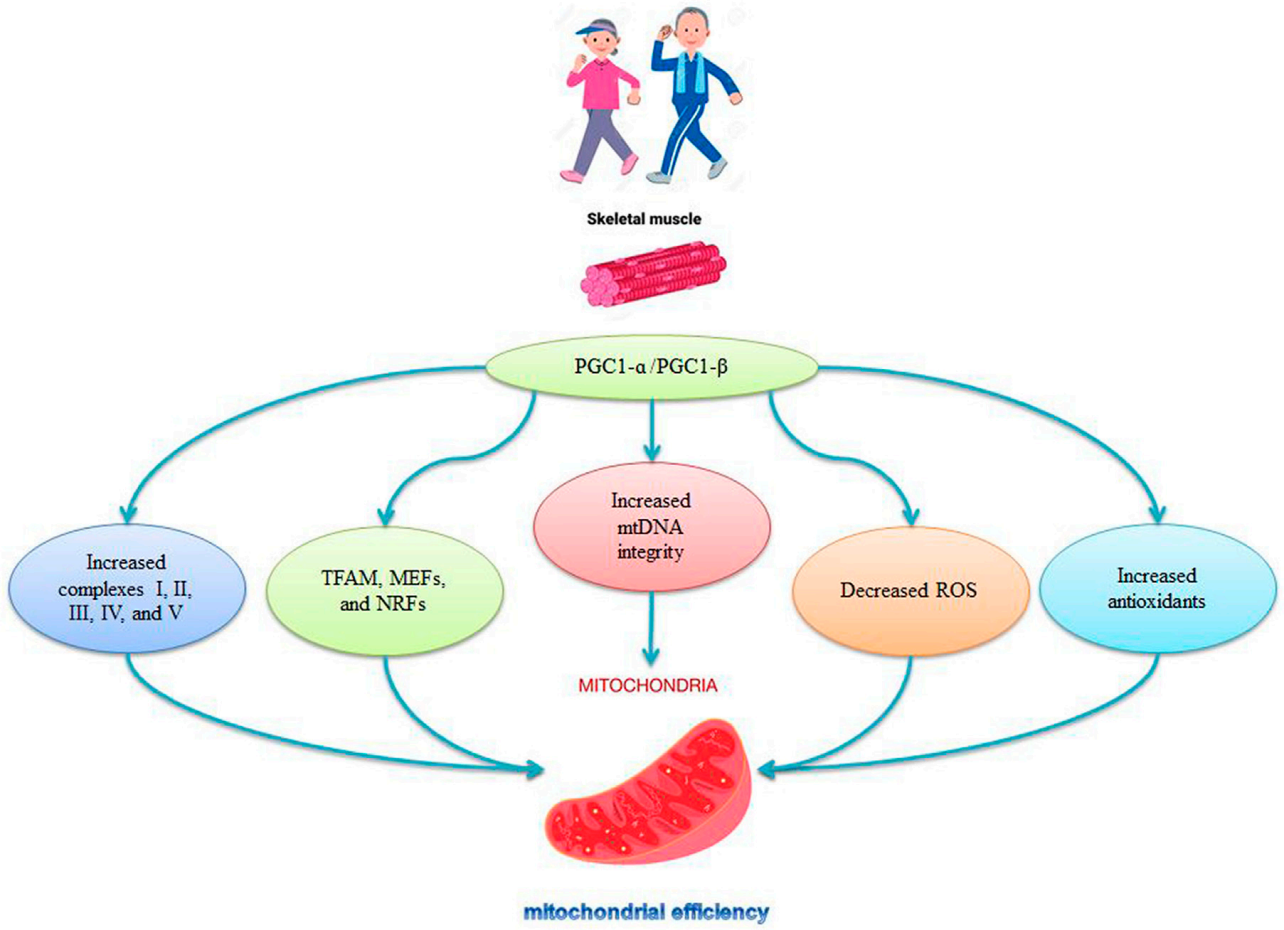
Role of PGC-1ɑ in mitochondrial regulation by exercise.

The expression levels of PGC-1α, PGC-1β, and TFAM in muscles decrease during aging (Romanello, 2021). There are also decreases in mitochondrial biogenesis (PGC-1α, Nrf-1, TFAM) and mitochondrial fission markers (Mfn2 and Opa1) in older mice, indicating impaired mitochondrial quality control with age (Huang et al., 2019; Liu et al., 2020). The specific deletion of PGC1-α causes premature aging in mice is characterized by fiber damage that also increases inflammatory markers and decreases mitochondrial function (Dozio et al., 2021; Romanello, 2021). The release of H_2_O_2_ and NO into the cytosol in older animals reduces mitochondrial biogenesis through PGC1-ɑ (López-Lluch et al., 2008), while delayed aging occurs in muscle-specific PGC1-α overexpressed mice (Romanello, 2021). The decreased levels of TFAM, NRF1, and PGC-1α activity during aging are reversed by aerobic exercise in aged mice (Harper et al., 2021) and older men and women (Harper et al., 2021) (Figure 3)*.* Exercise also increases PGC-1α, NRF1, and NRF2 mRNA (Baar et al., 2002; Ping et al., 2015), and is associated with NRF-1 genotypes and increased human aerobic capacity (He et al., 2008) (Figure 3)*.* Thus, activation of PGC-1α stimulates mitochondrial synthesis, followed by activation of NRF-1 and increases in TFAM synthesis and replication of mtDNA, leading to increases in the number of DNA copies and mitochondrial replication (Viña et al., 2009) (Figure 3). Mitochondrial synthesis is stimulated by the PGC-1α-NRF1-TFAM pathway, which is disrupted in aging; hence exercise, particularly aerobic exercise, activates mitochondrial synthesis in young and older animals (Viña et al., 2009).

### Exercise intensity and mitochondrial biogenesis

Intense exercise causes a faster and greater improvement in the mitochondrial respiratory capacity because high intensity (80% of VO_2max_) cycling engendered a 10.2-fold increase in PGC-1α mRNA, while low intensity (40% of VO_2max_) cycling produced only a 3.8-fold increase in PGC-1α mRNA (Harper et al., 2021). Training at 80% of VO_2max_ recruits primarily type II fibers, while training at 40% of VO_2max_ recruits primarily type I fibers (Harper et al., 2021). Training causes a greater upregulation of PGC-1α transcription in type II muscle fibers (Russell et al., 2003), because 6 weeks of interval training at 70%–80% of VO_2max_ produced a 2.8-fold increase in PGC-1α protein content in type IIa fibers and a 1.5-fold increase in type I (slow twitch) and type IIX (fast twitch) fibers (Harper et al., 2021). These findings suggest that more intense exercise protocols lead to greater increases in mitochondrial biogenesis genes in people with sarcopenia.

### Effects of exercise and sarcopenia on PGC-1α regulators

An important feature of aging-related sarcopenia is low metabolism and dysfunctional metabolism in skeletal muscle that accelerates aging (Liang et al., 2021). Age related decreases in muscle mass, muscle strength, and coordination are associated with reduced AMPK/PGC-1α signaling, while activation of AMPK/PGC-1α prevents skeletal muscle atrophy (Ko and Ko, 2021). Lifelong aerobic exercise can reduce protein degradation and improve mitochondrial quality control through AMPK/PGC-1α signaling pathways, and so delay sarcopenia (Liang et al., 2021). Acute exercise activates signals that converge on PGC-1α, leading to 1) activation of calcium-dependent protein kinase/calmodulin (CaMK) 2) activation of p38 MAPK, which is sensitive to multiple stressors such as ROS, and 3) phosphorylation of AMPK (Liang et al., 2021). Regular lifelong aerobic exercise promotes mitochondrial biogenesis by activating the AMPK/PGC-1α signaling pathway (Liao et al., 2017; Liang et al., 2021). Exercise increases intracellular calcium levels and creates an energy imbalance, stimulating mitochondrial biogenesis by activation of AMPK, CaMK, and MAPKs (Memme and Hood, 2021). Three kinases (AMPK, CaMKII, and MAPK) are upstream mediators of the activation of PGC-1α by physical activity for the positive regulation of PGC-1α (Wang et al., 2011; Margolis and Pasiakos, 2013; Pahlavani, 2022). These findings indicate that exercise increases intracellular energy metabolism, activates multiple intracellular stressors, and elevates intracellular calcium concentrations through PGC-1α signaling to induce mitochondrial biogenesis, proliferation, and differentiation of muscle cells by increasing the NRF1/2/TFAM and AKT/mTOR pathways.

## Conclusion

Sarcopenia describes decreases in skeletal muscle mass and functional decline that lead to a decrease in quality of life in the elderly (Liu et al., 2021) and which has several causes including such as inactivity, large decreases in antioxidant defenses, impaired mitochondrial mitophagy, and reduced mitochondrial biogenesis (Romanello, 2021). There is also poor coupling of ETC complexes and increased electron leakage during sarcopenia, causing oxidative stress (increases $O_{2}^{-}$ and H_2_O_2_), reduced mitochondrial function, increased deletion of mtDNA, and decreased mitochondrial enzyme activity (Harper et al., 2021). On the other hand, levels of antioxidants such as CAT, SOD, PRDXs, GPx, and GSH are reduced in aging muscles (Cesare et al., 2020).

The balance between fusion and fission, which is essential for maintaining effective mitochondrial function in skeletal muscle, is often compromised with age (Romanello, 2021) due to decreases in mitochondrial fission factors (Drp1, Fis1, and Mff) and mitochondrial fusion factors (Mfn1, Mfn2, and OPA1) in people with sarcopenia (Di Liu et al., 2021). In addition, reduced levels of mitophagy markers such as PINK1, Parkin, BNIP3, NIX, PHB2, FUNDC1, and AMBRA1 negatively impact sarcopenia-related muscle weakness and the quality of life in the elderly. Possible interventions for sarcopenia include exercise, gene therapy, dietary supplements, anabolic hormones, and increasing antioxidant capacity (Liang et al., 2021). Of this, exercise improves the number and function of mitochondria (Ferri et al., 2020) and the coupling of ETC complexes, reduces electron leakage, reduces oxidative stress, repairs mtDNA, increases mitochondrial enzyme activity, and improves mitochondrial efficiency (Cesare et al., 2020). Sarcopenia is also accompanied by impaired mitochondrial-related signals such as oxidative stress, antioxidant enzymes, and inflammatory factors leading to impaired mitochondrial dynamics, mitophagy, and mitochondria biogenesis. In addition, muscle aging decreases expression levels of PGC-1α, PGC-1β, TFAM, and Nrf-1 (Romanello, 2021), indicating impaired mitochondrial quality control during aging (Liu et al., 2020). Exercise activates upstream PGC-1α signals such as AMPK, CaMK, and MAPKs (p38 MAPK, ERK, JNK) that converge on PGC-1α, leading to increases in PGC-1β, TFAM, and Nrf-1 expression and mitochondrial biogenesis. Thus, exercise can be used as a therapeutic option to improve the quality and quantity of mitochondria.

## Future perspectives

Moderate-intensity exercise is a promising therapy to prevent sarcopenia due to its multitudinous effects (mitophagy effects, antioxidant defenses, mitochondrial biogenesis, mitochondrial dynamics, and PGC-1α regulators) on mitochondrial adaptations. While the benefits of exercise therapy along with nutritional supplements and gene therapy have not been investigated in large-scale clinical trials in people with sarcopenia. Moreover, only studies focusing on the effects of exercises affecting cardio-respiratory functions were explored and there is nothing or not much out about how weightlifting could affect mitochondrial mechanisms. In addition, it seems necessary to study the effect of exercise on other diseases. Hence, the study of the effect of exercise on the gender difference of mitochondria in the muscles of people with sarcopenia seems interesting to be conducted. Therefore, it seems that future research should be focused on these field.

## Glossary

**ADP**: Adenosine diphosphate
**ATP**: Adenosine triphosphate
**AMPK**: AMP-activated protein kinase
**AMBRA1**: activating molecule in BECN1 regulated autophagy protein 1
**BNI3**: Bcl2/adenovirus E1B 19 kDa protein-interacting protein 3
**CaMK**: calcium-dependent protein kinase/calmodulin
**CAT**: catalase
**COX**: Cytochrome-c oxidase
**complex V**: ATP synthase
**CS**: citrate synthase
**DLCs**: dynein light chains
**Drp1**: dynamin-related protein 1
**ER**: endoplasmic reticulum
**ERR**: estrogen-related receptor
**ETC**: electron transport chain
**FADH2**: flavin adenine dinucleotide
**Fis1**: fission protein 1
**FoxOs**: forkhead box
**GPx**: glutathione peroxidase
**GSH**: glutathione transferase
**H_2_O_2_**: hydrogen peroxide
**IMM**: inner mitochondrial membrane
**FUNDC1**: FUN14 domain containing 1
**LC3**: light chain 3
**ER**: endoplasmic reticulum
**MAM**: mitochondrial contact site
**Mff**: mitochondrial fission factor
**Mfn1**: mitofusin 1
**Mfn2**: mitofusin 2
**MRC**: mitochondrial respiratory chain
**mtDNA**: mitochondria DNA
**NADH**: nicotinamide adenine dinucleotide
**NF-κB**: kappa B nuclear factor
**NIX**: NIP3-like-protein X
**NRFs**: nuclear respiratory factors
$O_{2}^{-}$: superoxide anion
**•OH**: hydroxyl radicals
**OMM**: outer mitochondria membrane
**OPA1**: optic atrophy 1
**PGC-1α**: peroxisome proliferator-activated receptor-γ coactivator
**PKB or AKT**: Protein kinase B
**PHB2**: prohibitin 2
**PINK1**: PTEN-induced kinase 1
**PI3Ks**: Phosphoinositide 3-kinases
**poly-Ub**: poly-ubiquitin
**PPARs**: peroxisome proliferator-activated receptors
**PRDX**: Peroxiredoxin
**p38 MAPK**: mitogen-activated protein p38 kinase
**RNS**: reactive nitrogen species
**ROS**: reactive oxygen species
**SDH**: succinate dehydrogenase
**SOD**: superoxide dismutase
**TFAM**: transcriptional factor A mitochondrial
**TNF-ɑ**: tumor necrosis factor-α
**WHO**: World Health Organization

## References

[B1] Ahn B. Ranjit R. Piekarz K. Poopal A. Bian J. Sataranatarajan K. (2018). Skeletal muscle specific overexpression of the mitochondrial H2O2 scavenger, peroxiredoxin 3, rescues mitochondrial dysfunction and sarcopenia phenotypes elicited by redox imbalance. Free Radic. Biol. Med. 128, S123. 10.1016/j.freeradbiomed.2018.10.302

[B2] Alway S. E. Mohamed J. S. Myers M. J. (2017). Mitochondria initiate and regulate sarcopenia. Exerc. Sport Sci. Rev. 45 (2), 58–69. 10.1249/JES.0000000000000101 28098577 PMC5357179

[B3] Baar K. Wende A. R. Jones T. E. Marison M. Nolte L. A. Chen M. (2002). Adaptations of skeletal muscle to exercise: Rapid increase in the transcriptional coactivator PGC‐1. FASEB J. 16 (14), 1879–1886. 10.1096/fj.02-0367com 12468452

[B4] Balan E. Schwalm C. Naslain D. Nielens H. Francaux M. Deldicque L. (2019). Regular endurance exercise promotes fission, mitophagy, and oxidative phosphorylation in human skeletal muscle independently of age. Front. Physiol. 10, 1088. 10.3389/fphys.2019.01088 31507451 PMC6713923

[B5] Blottner D. Capitanio D. Trautmann G. Furlan S. Gambara G. Moriggi M. (2021). Nitrosative redox homeostasis and antioxidant response defense in disused vastus lateralis muscle in long-term bedrest (toulouse cocktail study). Antioxidants 10 (3), 378. 10.3390/antiox10030378 33802593 PMC8001160

[B6] Borges I. B. P. de Oliveira D. S. Marie S. K. N. Lenario A. M. Oba-Shinjo S. M. Shinjo S. K. (2021). Exercise training attenuates ubiquitin-proteasome pathway and increases the genes related to autophagy on the skeletal muscle of patients with inflammatory myopathies. J. Clin. Rheumatol. 27, S224–S231. 10.1097/rhu.0000000000001721 34227790

[B7] Brinkmann C. Chung N. Schmidt U. Kreutz T. Lenzen E. Schiffer T. (2012). Training alters the skeletal muscle antioxidative capacity in non‐insulin‐dependent type 2 diabetic men. Scand. J. Med. Sci. Sports 22 (4), 462–470. 10.1111/j.1600-0838.2010.01273.x 21477162

[B8] Brown L. A. Macpherson P. C. Koch L. G. Qi N. R. Britton S. L. Brooks S. V. (2019). Late life maintenance and enhancement of functional exercise capacity in low and high responding rats after low intensity treadmill training. Exp. Gerontol. 125, 110657. 10.1016/j.exger.2019.110657 31306740 PMC6707857

[B9] Caffin F. Prola A. Piquereau J. Novotova M. David D. Garnier A. (2013). Altered skeletal muscle mitochondrial biogenesis but improved endurance capacity in trained OPA1‐deficient mice. J. Physiol. 591 (23), 6017–6037. 10.1113/jphysiol.2013.263079 24042504 PMC3872767

[B10] Calvani R. Joseph A-M. Adhihetty P. J. Miccheli A. Bossola M. Leeuwenburgh C. (2013). Mitochondrial pathways in sarcopenia of aging and disuse muscle atrophy. Biol. Chem. 394 (3), 393–414. 10.1515/hsz-2012-0247 23154422 PMC3976204

[B11] Casuso R. A. Huertas J. R. (2020). The emerging role of skeletal muscle mitochondrial dynamics in exercise and ageing. Ageing Res. Rev. 58, 101025. 10.1016/j.arr.2020.101025 32018055

[B12] Ceci R. Duranti G. Di Filippo E. S. Bondi D. Verratti V. Doria C. (2020). Corrigendum to "Endurance training improves plasma superoxide dismutase activity in healthy elderly" [Mechanisms of Ageing and Development 185 (2020) 111190]. Mech. Ageing Dev. 185, 111214. 10.1016/j.mad.2020.111214 32081436

[B13] Cesare M. M. Felice F. Santini V. Di Stefano R. (2020). Antioxidants in sport sarcopenia. Nutrients 12 (9), 2869. 10.3390/nu12092869 32961753 PMC7551250

[B14] Chang Y. C. Chen Y. T. Liu H. W. Chan Y. C. Liu M. Y. Hu S. H. (2019). Oligonol alleviates sarcopenia by regulation of signaling pathways involved in protein turnover and mitochondrial quality. Mol. Nutr. Food Res. 63 (10), 1801102. 10.1002/mnfr.201801102 30793867

[B15] Chen P. B. Yang J. S. Park Y. (2018). Adaptations of skeletal muscle mitochondria to obesity, exercise, and polyunsaturated fatty acids. Lipids 53 (3), 271–278. 10.1002/lipd.12037 29663395

[B16] Cho B. J. Kim J. T. Lee Y. J. Yu S. H. Choi S. H. Lim S. (2008). Alteration of antioxidant enzyme gene expression with aging and exercise in Fisher rats. J. Korean Geriatrics Soc. 12 (3), 146–152.

[B17] Corsetto P. A. Montorfano G. Klersy C. Massimino L. Infantino V. Iannello G. (2019). Fatty acid profile and antioxidant status fingerprint in sarcopenic elderly patients: Role of diet and exercise. Nutrients 11 (11), 2569. 10.3390/nu11112569 31653011 PMC6893529

[B18] Cruz-Jentoft A. J. Romero-Yuste S. Carmona E. C. Nolla J. M. (2021). Sarcopenia, immune-mediated rheumatic diseases, and nutritional interventions. Aging Clin. Exp. Res. 33, 2929–2939. 10.1007/s40520-021-01800-7 33566325 PMC8595168

[B19] De Castro M. Cavalcanti Neto F. Lima L. Da Silva F. De Oliveira R. Zanesco A. (2009). Production of free radicals and catalase activity during acute exercise training in young men. Biol. Sport 26 (2), 113–118. 10.5604/20831862.890157

[B20] Di Bartolomeo S. Corazzari M. Nazio F. Oliverio S. Lisi G. Antonioli M. (2010). The dynamic interaction of AMBRA1 with the dynein motor complex regulates mammalian autophagy. J. Cell. Biol. 191 (1), 155–168. 10.1083/jcb.201002100 20921139 PMC2953445

[B21] Di Liu Y. B. Tao X. H. Pan W. L. Wu Y. X. Wang X. H. He Y. Q. (2021). Mitochondrial quality control in sarcopenia: Updated overview of mechanisms and interventions. Aging Dis. 12, 2016–2030. 10.14336/AD.2021.0427 34881083 PMC8612607

[B22] Ding W-X. Yin X-M. (2012). Mitophagy: Mechanisms, pathophysiological roles, and analysis. Biol. Chem. 393 (7), 547–564. 10.1515/hsz-2012-0119 22944659 PMC3630798

[B23] Dorn G. W. (2010). Mitochondrial pruning by nix and BNip3: An essential function for cardiac-expressed death factors. J. Cardiovasc. Transl. Res. 3 (4), 374–383. 10.1007/s12265-010-9174-x 20559783 PMC2900478

[B24] Dozio E. Vettoretti S. Lungarella G. Messa P. Corsi Romanelli M. M. (2021). Sarcopenia in chronic kidney disease: Focus on advanced glycation end products as mediators and markers of oxidative stress. Biomedicines 9 (4), 405. 10.3390/biomedicines9040405 33918767 PMC8068965

[B25] Drake J. C. Wilson R. J. Yan Z. (2016). Molecular mechanisms for mitochondrial adaptation to exercise training in skeletal muscle. FASEB J. 30 (1), 13–22. 10.1096/fj.15-276337 26370848 PMC6137621

[B26] Drummond M. J. Addison O. Brunker L. Hopkins P. N. McClain D. A. LaStayo P. C. (2014). Downregulation of E3 ubiquitin ligases and mitophagy-related genes in skeletal muscle of physically inactive, frail older women: A cross-sectional comparison. J. Gerontol. A Biol. Sci. Med. Sci. 69 (8), 1040–1048. 10.1093/gerona/glu004 24526667 PMC4111292

[B27] Eshima H. Siripoksup P. Mahmassani Z. S. Johnson J. M. Ferrara P. J. Verkerke A. R. (2020). Neutralizing mitochondrial ROS does not rescue muscle atrophy induced by hindlimb unloading in female mice. J. Appl. Physiol. 129 (1), 124–132. 10.1152/japplphysiol.00456.2019 32552434 PMC7469234

[B28] Fang W. Li Z. Liu Z. Feng H. (2020). Changes of mitochondrial respiratory function and PHB1 expression in rat skeletal muscle after moderate-intensity training. Chin. J. Tissue Eng. Res. 24 (8), 1207.

[B29] Ferri E. Marzetti E. Calvani R. Picca A. Cesari M. Arosio B. (2020). Role of age-related mitochondrial dysfunction in sarcopenia. Int. J. Mol. Sci. 21 (15), 5236. 10.3390/ijms21155236 32718064 PMC7432902

[B30] Fimia G. Corazzari M. Antonioli M. Piacentini M. (2013). Ambra1 at the crossroad between autophagy and cell death. Oncogene 32 (28), 3311–3318. 10.1038/onc.2012.455 23069654

[B31] Fimia G. M. Stoykova A. Romagnoli A. Giunta L. Di Bartolomeo S. Nardacci R. (2007). Ambra1 regulates autophagy and development of the nervous system. Nature 447 (7148), 1121–1125. 10.1038/nature05925 17589504

[B32] Fu T. Xu Z. Liu L. Guo Q. Wu H. Liang X. (2018). Mitophagy directs muscle-adipose crosstalk to alleviate dietary obesity. Cell. Rep. 23 (5), 1357–1372. 10.1016/j.celrep.2018.03.127 29719250

[B33] Galli D. Carubbi C. Masselli E. Vaccarezza M. Presta V. Pozzi G. (2021). Physical activity and redox balance in the elderly: Signal transduction mechanisms. Appl. Sci. 11 (5), 2228. 10.3390/app11052228

[B34] Gan Z. Fu T. Kelly D. P. Vega R. B. (2018). Skeletal muscle mitochondrial remodeling in exercise and diseases. Cell. Res. 28 (10), 969–980. 10.1038/s41422-018-0078-7 30108290 PMC6170448

[B35] Gao J. Yu L. Wang Z. Wang R. Liu X. (2020). Induction of mitophagy in C2C12 cells by electrical pulse stimulation involves increasing the level of the mitochondrial receptor FUNDC1 through the AMPK-ULK1 pathway. Am. J. Transl. Res. 12 (10), 6879–6894. 33194079 PMC7653589

[B36] Gruber J. Chen C-B. Fong S. Ng L. F. Teo E. Halliwell B. (2015). *Caenorhabditis elegans*: What we can and cannot learn from aging worms. Antioxid. Redox Signal. 23 (3), 256–279. 10.1089/ars.2014.6210 25544992

[B37] Grumati P. Coletto L. Schiavinato A. Castagnaro S. Bertaggia E. Sandri M. (2011). Physical exercise stimulates autophagy in normal skeletal muscles but is detrimental for collagen VI-deficient muscles. Autophagy 7 (12), 1415–1423. 10.4161/auto.7.12.17877 22024752 PMC3288016

[B38] Gu Q. Wang B. Zhang X-F. Ma Y-P. Liu J-D. Wang X-Z. (2014). Chronic aerobic exercise training attenuates aortic stiffening and endothelial dysfunction through preserving aortic mitochondrial function in aged rats. Exp. Gerontol. 56, 37–44. 10.1016/j.exger.2014.02.014 24607516

[B39] Gu W. Wan D. Qian Q. Yi B. He Z. Gu Y. (2014). Ambra1 is an essential regulator of autophagy and apoptosis in SW620 cells: Pro-survival role of Ambra1. PLoS One 9 (2), e90151. 10.1371/journal.pone.0090151 24587252 PMC3936000

[B40] Guan Y. Drake J. C. Yan Z. (2019). Exercise-induced mitophagy in skeletal muscle and heart. Exerc. Sport Sci. Rev. 47 (3), 151–156. 10.1249/JES.0000000000000192 30985475 PMC6579614

[B41] Guescini M. Tiano L. Genova M. L. Polidori E. Silvestri S. Orlando P. (2017). The combination of physical exercise with muscle-directed antioxidants to counteract sarcopenia: A biomedical rationale for pleiotropic treatment with creatine and coenzyme Q10. Oxid. Med. Cell. Longev. 2017, 7083049. 10.1155/2017/7083049 29123615 PMC5632475

[B42] Gusdon A. M. Callio J. Distefano G. O'Doherty R. M. Goodpaster B. H. Coen P. M. (2017). Exercise increases mitochondrial complex I activity and DRP1 expression in the brains of aged mice. Exp. Gerontol. 90, 1–13. 10.1016/j.exger.2017.01.013 28108329 PMC5346470

[B43] Harper C. Gopalan V. Goh J. (2021). Exercise rescues mitochondrial coupling in aged skeletal muscle: A comparison of different modalities in preventing sarcopenia. J. Transl. Med. 19 (1), 71–17. 10.1186/s12967-021-02737-1 33593349 PMC7885447

[B44] He Z. Hu Y. Feng L. Li Y. Liu G. Xi Y. (2008). NRF-1 genotypes and endurance exercise capacity in young Chinese men. Br. J. Sports Med. 42 (5), 361–366. 10.1136/bjsm.2007.042945 18184751

[B45] Hepple R. T. (2014). Mitochondrial involvement and impact in aging skeletal muscle. Front. Aging Neurosci. 6, 211. 10.3389/fnagi.2014.00211 25309422 PMC4159998

[B46] Hoffman N. J. Parker B. L. Chaudhuri R. Fisher-Wellman K. H. Kleinert M. Humphrey S. J. (2015). Global phosphoproteomic analysis of human skeletal muscle reveals a network of exercise-regulated kinases and AMPK substrates. Cell. Metab. 22 (5), 922–935. 10.1016/j.cmet.2015.09.001 26437602 PMC4635038

[B47] Homma T. Kurahashi T. Lee J. Nabeshima A. Yamada S. Fujii J. (2018). Double knockout of peroxiredoxin 4 (Prdx4) and superoxide dismutase 1 (Sod1) in mice results in severe liver failure. Oxid. Med. Cell. Longev. 2018, 2812904. 10.1155/2018/2812904 30050648 PMC6040270

[B48] Huang D-D. Fan S-D. Chen X-Y. Yan X-L. Zhang X-Z. Ma B-W. (2019). Nrf2 deficiency exacerbates frailty and sarcopenia by impairing skeletal muscle mitochondrial biogenesis and dynamics in an age-dependent manner. Exp. Gerontol. 119, 61–73. 10.1016/j.exger.2019.01.022 30690066

[B49] Hussey S. E. Sharoff C. G. Garnham A. Zhengping Y. Bowen B. P. Mandarino L. J. (2013). Effect of exercise on the skeletal muscle proteome in patients with type 2 diabetes. Med. Sci. Sports Exerc. 45 (6), 1069–1076. 10.1249/MSS.0b013e3182814917 23274603 PMC3660427

[B50] Jackson M. J. (2016). Reactive oxygen species in sarcopenia: Should we focus on excess oxidative damage or defective redox signalling? Mol. Asp. Med. 50, 33–40. 10.1016/j.mam.2016.05.002 27161871

[B51] Jamart C. Naslain D. Gilson H. Francaux M. (2013). Higher activation of autophagy in skeletal muscle of mice during endurance exercise in the fasted state. Am. J. Physiol. Endocrinol. Metab. 305, E964–E974. 10.1152/ajpendo.00270.2013 23964069

[B52] Jensen G. L. (2008). Inflammation: Roles in aging and sarcopenia. JPEN. J. Parenter. Enter. Nutr. 32 (6), 656–659. 10.1177/0148607108324585 18974248

[B53] Ji L. L. (2002). Exercise‐induced modulation of antioxidant defense. Ann. N. Y. Acad. Sci. 959 (1), 82–92. 10.1111/j.1749-6632.2002.tb02085.x 11976188

[B54] Jiang X. Cai S. Jin Y. Wu F. He J. Wu X. (2021). Irisin attenuates oxidative stress, mitochondrial dysfunction, and apoptosis in the H9C2 cellular model of septic cardiomyopathy through augmenting fundc1-dependent mitophagy. Oxid. Med. Cell. Longev. 2021, 2989974. 10.1155/2021/2989974 34457111 PMC8390168

[B55] Joseph A-M. Adhihetty P. J. Wawrzyniak N. R. Wohlgemuth S. E. Picca A. Kujoth G. C. (2013). Dysregulation of mitochondrial quality control processes contribute to sarcopenia in a mouse model of premature aging. PLoS One 8 (7), e69327. 10.1371/journal.pone.0069327 23935986 PMC3720551

[B56] Kadoguchi T. Shimada K. Miyazaki T. Kitamura K. Kunimoto M. Aikawa T. (2020). Promotion of oxidative stress is associated with mitochondrial dysfunction and muscle atrophy in aging mice. Geriatr. Gerontol. Int. 20 (1), 78–84. 10.1111/ggi.13818 31758637

[B57] Kim Y. A. Kim Y. S. Oh S. L. Kim H-J. Song W. (2013). Autophagic response to exercise training in skeletal muscle with age. J. Physiol. Biochem. 69 (4), 697–705. 10.1007/s13105-013-0246-7 23471597

[B58] Ko Y. J. Ko I-G. (2021). Voluntary wheel running exercise improves aging-induced sarcopenia via activation of peroxisome proliferator-activated receptor gamma coactivator-1α/fibronectin type III domain-containing protein 5/adenosine monophosphate-activated protein kinase signaling pathway. Int. Neurourol. J. 25 (1), S27–S34. 10.5213/inj.2142170.085 34053208 PMC8171240

[B59] Laker R. C. Drake J. C. Wilson R. J. Lira V. A. Lewellen B. M. Ryall K. A. (2017). Ampk phosphorylation of Ulk1 is required for targeting of mitochondria to lysosomes in exercise-induced mitophagy. Nat. Commun. 8 (1), 548–613. 10.1038/s41467-017-00520-9 28916822 PMC5601463

[B60] Lambertucci R. H. Levada-Pires A. C. Rossoni L. V. Curi R. Pithon-Curi T. C. (2007). Effects of aerobic exercise training on antioxidant enzyme activities and mRNA levels in soleus muscle from young and aged rats. Mech. Ageing Dev. 128 (3), 267–275. 10.1016/j.mad.2006.12.006 17224177

[B61] Lampert M. A. Orogo A. M. Najor R. H. Hammerling B. C. Leon L. J. Wang B. J. (2019). BNIP3L/NIX and FUNDC1-mediated mitophagy is required for mitochondrial network remodeling during cardiac progenitor cell differentiation. Autophagy 15 (7), 1182–1198. 10.1080/15548627.2019.1580095 30741592 PMC6613840

[B62] Lee K-P. Shin Y. J. Cho S. C. Lee S-M. Bahn Y. J. Kim J. Y. (2014). Peroxiredoxin 3 has a crucial role in the contractile function of skeletal muscle by regulating mitochondrial homeostasis. Free Radic. Biol. Med. 77, 298–306. 10.1016/j.freeradbiomed.2014.09.010 25224038

[B63] Lenhare L. Crisol B. M. Silva V. R. Katashima C. K. Cordeiro A. V. Pereira K. D. (2017). Physical exercise increases Sestrin 2 protein levels and induces autophagy in the skeletal muscle of old mice. Exp. Gerontol. 97, 17–21. 10.1016/j.exger.2017.07.009 28729213

[B64] Li D. Lai Y. Yue Y. Rabinovitch P. S. Hakim C. Duan D. (2009). Ectopic catalase expression in mitochondria by adeno-associated virus enhances exercise performance in mice. PLoS One 4 (8), e6673. 10.1371/journal.pone.0006673 19690612 PMC2723912

[B65] Liang J. Zeng Z. Zhang Y. Chen N. (2020). Regulatory role of exercise-induced autophagy for sarcopenia. Exp. Gerontol. 130, 110789. 10.1016/j.exger.2019.110789 31765742

[B66] Liang J. Zhang H. Zeng Z. Wu L. Zhang Y. Guo Y. (2021). Lifelong aerobic exercise alleviates sarcopenia by activating autophagy and inhibiting protein degradation via the AMPK/PGC-1α signaling pathway. Metabolites 11 (5), 323. 10.3390/metabo11050323 34069829 PMC8157243

[B67] Liao Z. Y. Chen J. L. Xiao M. H. Sun Y. Zhao Y. X. Pu D. (2017). The effect of exercise, resveratrol or their combination on Sarcopenia in aged rats via regulation of AMPK/Sirt1 pathway. Exp. Gerontol. 98, 177–183. 10.1016/j.exger.2017.08.032 28847722

[B68] Lightfoot A. P. Cooper R. G. (2016). Endurance exercise: An important therapeutic adjuvant in the overall treatment of myositis? Arthritis Rheumatol. 68 (7), 1578–1581. 10.1002/art.39615 26866277

[B69] Liu H-W. Chang Y-C. Chan Y-C. Hu S-H. Liu M-Y. Chang S-J. (2020). Dysregulations of mitochondrial quality control and autophagic flux at an early age lead to progression of sarcopenia in SAMP8 mice. Biogerontology 21 (3), 367–380. 10.1007/s10522-020-09867-x 32130580

[B70] Liu S. Yu C. Xie L. Niu Y. Fu L. (2021). Aerobic exercise improves mitochondrial function in sarcopenia mice through Sestrin2 in an AMPKα2-dependent manner. J. Gerontol. A Biol. Sci. Med. Sci. 76 (7), 1161–1168. 10.1093/gerona/glab029 33512470

[B71] Lo J. H. Yiu T. Ong M. T. Lee W. Y. U K. P. (2020). Sarcopenia: Current treatments and new regenerative therapeutic approaches. J. Orthop. Transl. 23, 38–52. 10.1016/j.jot.2020.04.002 PMC7256062 32489859

[B72] López-Lluch G. Irusta P. M. Navas P. de Cabo R. (2008). Mitochondrial biogenesis and healthy aging. Exp. Gerontol. 43 (9), 813–819. 10.1016/j.exger.2008.06.014 18662766 PMC2562606

[B73] López-Lluch G. (2017). Mitochondrial activity and dynamics changes regarding metabolism in ageing and obesity. Mech. Ageing Dev. 162, 108–121. 10.1016/j.mad.2016.12.005 27993601

[B74] Manganelli V. Matarrese P. Antonioli M. Gambardella L. Vescovo T. Gretzmeier C. (2020). Raft-like lipid microdomains drive autophagy initiation via AMBRA1-ERLIN1 molecular association within MAMs. Autophagy 17, 2528–2548. 10.1080/15548627.2020.1834207 33034545 PMC8496542

[B75] Margolis L. M. Pasiakos S. M. (2013). Optimizing intramuscular adaptations to aerobic exercise: Effects of carbohydrate restriction and protein supplementation on mitochondrial biogenesis. Adv. Nutr. 4 (6), 657–664. 10.3945/an.113.004572 24228194 PMC3823511

[B76] Marzetti E. Lawler J. M. Hiona A. Manini T. Seo A. Y. Leeuwenburgh C. (2008). Modulation of age-induced apoptotic signaling and cellular remodeling by exercise and calorie restriction in skeletal muscle. Free Radic. Biol. Med. 44 (2), 160–168. 10.1016/j.freeradbiomed.2007.05.028 18191752

[B77] Mason S. A. Trewin A. J. Parker L. Wadley G. D. (2020). Antioxidant supplements and endurance exercise: Current evidence and mechanistic insights. Redox Biol. 35, 101471. 10.1016/j.redox.2020.101471 32127289 PMC7284926

[B78] Matta L. Fonseca T. S. Faria C. C. Lima-Junior N. C. De Oliveira D. F. Maciel L. (2021). The effect of acute aerobic exercise on redox homeostasis and mitochondrial function of rat white adipose tissue. Oxid. Med. Cell. Longev. 2021, 4593496. 10.1155/2021/4593496 33603946 PMC7868166

[B79] Memme J. M. Erlich A. T. Phukan G. Hood D. A. (2021). Exercise and mitochondrial health. J. Physiol. 599 (3), 803–817. 10.1113/JP278853 31674658

[B80] Memme J. M. Hood D. A. (2021). Molecular basis for the therapeutic effects of exercise on mitochondrial defects. Front. Physiol. 11, 615038. 10.3389/fphys.2020.615038 33584337 PMC7874077

[B81] Mendes S. V. D. de Albuquerque I. B. Montenegro M. F. dos Santos C. A. A. de Serpa G. L. de Oliveira A. C. (2021). Efeitos de diferentes volumes de exercício físico aeróbio sobre a atividade da enzima catalase em diferentes tipos de fibras do músculo esquelético em ratos Wistar/Effects of different volumes of aerobic exercise on catalase enzyme activity in different types of skeletal muscle fibers in Wistar rats. Braz. J. Dev. 7 (4), 41348–41356. 10.34117/bjdv7n4-546

[B82] Migliavacca E. Tay S. K. Patel H. P. Sonntag T. Civiletto G. McFarlane C. (2019). Mitochondrial oxidative capacity and NAD+ biosynthesis are reduced in human sarcopenia across ethnicities. Nat. Commun. 10 (1), 5808–5814. 10.1038/s41467-019-13694-1 31862890 PMC6925228

[B83] Moreira O. C. Estébanez B. Martínez-Florez S. Paz J. A. Cuevas M. J. González-Gallego J. (2017). Mitochondrial function and mitophagy in the elderly: Effects of exercise. Oxidative Med. Cell. Longev. 2017, 2012798. 10.1155/2017/2012798 PMC5576425 28900532

[B84] Musumeci G. (2017). Sarcopenia and exercise “the state of the art”. J. Funct. Morphol. Kinesiol. 2 (4), 40. 10.3390/jfmk2040040

[B85] Ogura Y. Iemitsu M. Naito H. Kakigi R. Kakehashi C. Maeda S. (2011). Single bout of running exercise changes LC3-II expression in rat cardiac muscle. Biochem. Biophys. Res. Commun. 414 (4), 756–760. 10.1016/j.bbrc.2011.09.152 22005460

[B86] Organization W. H. (2020). WHO clinical consortium on healthy ageing 2019: Report of consortium meeting held 21-22 november 2019. Geneva, Switzerland: WHO.

[B87] Pacifici F. Della-Morte D. Piermarini F. Arriga R. Scioli M. G. Capuani B. (2020). Prdx6 plays a main role in the crosstalk between aging and metabolic sarcopenia. Antioxidants 9 (4), 329. 10.3390/antiox9040329 32316601 PMC7222359

[B88] Pahlavani H. A. (2022). Exercise therapy for people with sarcopenic obesity: Myokines and adipokines as effective actors. Front. Endocrinol. 13, 811751. 10.3389/fendo.2022.811751 PMC8892203 35250869

[B89] Parise G. Brose A. N. Tarnopolsky M. A. (2005). Resistance exercise training decreases oxidative damage to DNA and increases cytochrome oxidase activity in older adults. Exp. Gerontol. 40 (3), 173–180. 10.1016/j.exger.2004.09.002 15763394

[B90] Parry H. A. Roberts M. D. Kavazis A. N. (2020). Human skeletal muscle mitochondrial adaptations following resistance exercise training. Int. J. Sports Med. 41 (06), 349–359. 10.1055/a-1121-7851 32162291

[B91] Pascual-Fernández J. Fernández-Montero A. Córdova-Martínez A. Pastor D. Martínez-Rodríguez A. Sarcopenia Roche E. (2020). Sarcopenia: Molecular pathways and potential targets for intervention. Int. J. Mol. Sci. 21 (22), 8844. 10.3390/ijms21228844 33266508 PMC7700275

[B92] Phu S. Boersma D. Duque G. (2015). Exercise and sarcopenia. J. Clin. Densitom. 18 (4), 488–492. 10.1016/j.jocd.2015.04.011 26071171

[B93] Picca A. Calvani R. Lorenzi M. Menghi A. Galli M. Vitiello R. (2017). Mitochondrial dynamics signaling is shifted toward fusion in muscles of very old hip-fractured patients: Results from the Sarcopenia in HIp FracTure (SHIFT) exploratory study. Exp. Gerontol. 96, 63–67. 10.1016/j.exger.2017.06.005 28602957

[B94] Picca A. Calvani R. (2021). Molecular mechanism and pathogenesis of sarcopenia: An overview. Int. J. Mol. Sci. 22 (6), 3032. 10.3390/ijms22063032 33809723 PMC8002369

[B95] Ping Z. Zhang L. F. Cui Y. F. Chang Y. M. Jiang C. W. Meng Z. Z. (2015). The protective effects of salidroside from exhaustive exercise-induced heart injury by enhancing the PGC-1α–NRF1/NRF2 pathway and mitochondrial respiratory function in rats. Oxid. Med. Cell. Longev. 2015, 876825. 10.1155/2015/876825 26167242 PMC4488012

[B96] Rędowicz J. (2021). Session 9: Muscle and beyond. Acta Biochim. Pol. 68 (S1), 66–78.

[B97] Romanello V. (2021). The interplay between mitochondrial morphology and myomitokines in aging sarcopenia. Int. J. Mol. Sci. 22 (1), 91. 10.3390/ijms22010091 PMC7796142 33374852

[B98] Russell A. P. Feilchenfeldt J. Schreiber S. Praz M. Crettenand A. Gobelet C. (2003). Endurance training in humans leads to fiber type-specific increases in levels of peroxisome proliferator-activated receptor-γ coactivator-1 and peroxisome proliferator-activated receptor-α in skeletal muscle. Diabetes 52 (12), 2874–2881. 10.2337/diabetes.52.12.2874 14633846

[B99] Sánchez‐Castellano C. Martín‐Aragón S. Bermejo‐Bescós P. Vaquero‐Pinto N. Miret‐Corchado C. Merello de Miguel A. (2020). Biomarkers of sarcopenia in very old patients with hip fracture. J. Cachexia Sarcopenia Muscle 11 (2), 478–486. 10.1002/jcsm.12508 31912666 PMC7113494

[B100] Santos-Alves E. Marques-Aleixo I. Rizo-Roca D. Torrella J. Oliveira P. Magalhães J. (2015). Exercise modulates liver cellular and mitochondrial proteins related to quality control signaling. Life Sci. 135, 124–130. 10.1016/j.lfs.2015.06.007 26135624

[B101] Sataranatarajan K. Pharaoh G. Brown J. L. Ranjit R. Piekarz K. M. Street K. (2020). Molecular changes in transcription and metabolic pathways underlying muscle atrophy in the CuZnSOD null mouse model of sarcopenia. Geroscience 42 (4), 1101–1118. 10.1007/s11357-020-00189-x 32394347 PMC7394980

[B102] Schoenherr C. Byron A. Griffith B. Loftus A. Wills J. C. Munro A. F. (2020). The autophagy protein Ambra1 regulates gene expression by supporting novel transcriptional complexes. J. Biol. Chem. 295 (34), 12045–12057. 10.1074/jbc.RA120.012565 32616651 PMC7443501

[B103] Selsby J. T. (2011). Increased catalase expression improves muscle function in mdx mice. Exp. Physiol. 96 (2), 194–202. 10.1113/expphysiol.2010.054379 21041317 PMC4519830

[B104] Seo D. Y. Hwang B. G. (2020). Effects of exercise training on the biochemical pathways associated with sarcopenia. Phys. Act. Nutr. 24 (3), 32–38. 10.20463/pan.2020.0019 33108716 PMC7669465

[B105] Serra A. J. Pinto J. R. Prokić M. D. Arsa G. Vasconsuelo A. (2020). Oxidative stress in muscle diseases: Current and future therapy 2019. London, Hindawi: Hindawi. 10.1155/2020/6030417 PMC7193296 32377303

[B106] Shahar S. Kamaruddin N. S. Badrasawi M. Sakian N. I. M. Abd Manaf Z. Yassin Z. (2013). Effectiveness of exercise and protein supplementation intervention on body composition, functional fitness, and oxidative stress among elderly Malays with sarcopenia. Clin. Interv. Aging 8, 1365–1375. 10.2147/CIA.S46826 24143082 PMC3797615

[B107] Signorile A. Sgaramella G. Bellomo F. De Rasmo D. (2019). Prohibitins: A critical role in mitochondrial functions and implication in diseases. Cells 8 (1), 71. 10.3390/cells8010071 30669391 PMC6356732

[B108] Siu P. M. Bryner R. W. Martyn J. K. Alway S. E. (2004). Apoptotic adaptations from exercise training in skeletal and cardiac muscles. FASEB J. 18 (10), 1150–1152. 10.1096/fj.03-1291fje 15132982

[B109] Strappazzon F. Di Rita A. Peschiaroli A. Leoncini P. P. Locatelli F. Melino G. (2020). HUWE1 controls MCL1 stability to unleash AMBRA1-induced mitophagy. Cell. Death Differ. 27 (4), 1155–1168. 10.1038/s41418-019-0404-8 31434979 PMC7206129

[B110] Sullivan-Gunn M. J. Lewandowski P. A. (2013). Elevated hydrogen peroxide and decreased catalase and glutathione peroxidase protection are associated with aging sarcopenia. BMC Geriatr. 13 (1), 104–109. 10.1186/1471-2318-13-104 24093947 PMC3853025

[B111] Sun B. hong Wang J. yuan Lv Y. shu Zhu S. Yang J. zheng Ma J. (2008). Proteomic adaptation to chronic high intensity swimming training in the rat heart. Comp. Biochem. Physiol. Part D. Genomics Proteomics 3 (1), 108–117. 10.1016/j.cbd.2007.11.001 20483212

[B112] Teixeira A. Dias V. Pase C. Roversi K. Boufleur N. Barcelos R. (2012). Could dietary trans fatty acids induce movement disorders? Effects of exercise and its influence on Na+ K+-ATPase and catalase activity in rat striatum. Behav. Brain Res. 226 (2), 504–510. 10.1016/j.bbr.2011.10.005 22004982

[B113] Trewin A. J. Parker L. Shaw C. S. Hiam D. S. Garnham A. Levinger I. (2018). Acute HIIE elicits similar changes in human skeletal muscle mitochondrial H2O2 release, respiration, and cell signaling as endurance exercise even with less work. Am. J. Physiol. Regul. Integr. Comp. Physiol. 315 (5), R1003–R16. 10.1152/ajpregu.00096.2018 30183338

[B114] Triolo M. Hood D. A. (2021). Manifestations of age on autophagy, mitophagy and lysosomes in skeletal muscle. Cells 10 (5), 1054. 10.3390/cells10051054 33946883 PMC8146406

[B115] Van Remmen H. Pharaoh G. Ahn B. (2019). The role of mitochondrial peroxide release in the mechanisms underlying age‐related sarcopenia. FASEB J. 33 (S1), 342–343. 10.1096/fasebj.2019.33.1_supplement.342.3

[B116] Vilela T. C. Effting P. S. dos Santos Pedroso G. Farias H. Paganini L. Sorato H. R. (2018). Aerobic and strength training induce changes in oxidative stress parameters and elicit modifications of various cellular components in skeletal muscle of aged rats. Exp. Gerontol. 106, 21–27. 10.1016/j.exger.2018.02.014 29471131

[B117] Viña J. Gomez-Cabrera M. C. Borras C. Froio T. Sanchis-Gomar F. Martinez-Bello V. E. (2009). Mitochondrial biogenesis in exercise and in ageing. Adv. Drug Deliv. Rev. 61 (14), 1369–1374. 10.1016/j.addr.2009.06.006 19716394

[B118] Votion D. M. Fraipont A. Goachet A-G. Robert C. Van Erck E. Amory H. (2010). Alterations in mitochondrial respiratory function in response to endurance training and endurance racing. Equine Vet. J. 42, 268–274. 10.1111/j.2042-3306.2010.00271.x 21059017

[B119] Wadley A. J. Aldred S. Coles S. J. (2016). An unexplored role for Peroxiredoxin in exercise-induced redox signalling? Redox Biol. 8, 51–58. 10.1016/j.redox.2015.10.003 26748042 PMC4712319

[B120] Waltz T. B. Fivenson E. M. Morevati M. Li C. Becker K. G. Bohr V. A. (2018). Sarcopenia, aging and prospective interventional strategies. Curr. Med. Chem. 25 (40), 5588–5596. 10.2174/0929867324666170801095850 28762310 PMC5792375

[B121] Wang L. Mascher H. Psilander N. Blomstrand E. Sahlin K. (2011). Resistance exercise enhances the molecular signaling of mitochondrial biogenesis induced by endurance exercise in human skeletal muscle. J. Appl. Physiol. 111 (5), 1335–1344. 10.1152/japplphysiol.00086.2011 21836044

[B122] Wei Y. Chiang W-C. Sumpter R. Jr Mishra P. Levine B. (2017). Prohibitin 2 is an inner mitochondrial membrane mitophagy receptor. Cell. 168 (1-2), 224–238. e10. 10.1016/j.cell.2016.11.042 28017329 PMC5235968

[B123] Xu H. Ranjit R. Richardson A. Van Remmen H. (2021). Muscle mitochondrial catalase expression prevents neuromuscular junction disruption, atrophy, and weakness in a mouse model of accelerated sarcopenia. J. Cachexia Sarcopenia Muscle 12, 1582–1596. 10.1002/jcsm.12768 34559475 PMC8718066

[B124] Yan C. Gong L. Chen L. Xu M. Abou-Hamdan H. Tang M. (2020). PHB2 (prohibitin 2) promotes PINK1-PRKN/Parkin-dependent mitophagy by the PARL-PGAM5-PINK1 axis. Autophagy 16 (3), 419–434. 10.1080/15548627.2019.1628520 31177901 PMC6999623

[B125] Yao R-Q. Ren C. Xia Z-F. Yao Y-M. (2021). Organelle-specific autophagy in inflammatory diseases: A potential therapeutic target underlying the quality control of multiple organelles. Autophagy 17 (2), 385–401. 10.1080/15548627.2020.1725377 32048886 PMC8007140

[B126] Yoo S-Z. No M-H. Heo J-W. Park D-H. Kang J-H. Kim S. H. (2018). Role of exercise in age-related sarcopenia. J. Exerc. Rehabil. 14 (4), 551–558. 10.12965/jer.1836268.134 30276173 PMC6165967

[B127] Zhang J. Ney P. A. (2009). Role of BNIP3 and NIX in cell death, autophagy, and mitophagy. Cell. Death Differ. 16 (7), 939–946. 10.1038/cdd.2009.16 19229244 PMC2768230

[B128] Zhao D. Sun Y. Tan Y. Zhang Z. Hou Z. Gao C. (2018). Short-duration swimming exercise after myocardial infarction attenuates cardiac dysfunction and regulates mitochondrial quality control in aged mice. Oxidative Med. Cell. Longev. 2018, 4079041. 10.1155/2018/4079041 PMC5925211 29849892

[B129] Zhao Y. Zhu Q. Song W. Gao B. (2018). Exercise training and dietary restriction affect PINK1/Parkin and Bnip3/Nix-mediated cardiac mitophagy in mice. Gen. Physiol. Biophys. 37 (6), 657–666. 10.4149/gpb_2018020 30431438

